# Metabolomic adaptations and genetic polymorphism in ecopopulations of *Rhodiola linearifolia* Boriss

**DOI:** 10.3389/fpls.2025.1570411

**Published:** 2025-06-19

**Authors:** Malika Yerbay, Oxana N. Khapilina, Ainur S. Turzhanova, Irina G. Otradnykh, Irina A. Sedina, Aigerim Mamirova, Nazym K. Korbozova, Saule Magzumova, Kazhybek Ashimuly, Nataliya O. Kudrina, Vitaly G. Salnikov, Nina V. Terletskaya

**Affiliations:** ^1^ Faculty of Biology and Biotechnology, Al-Farabi Kazakh National University, Almaty, Kazakhstan; ^2^ Institute of Genetic and Physiology, Almaty, Kazakhstan; ^3^ National Center for Biotechnology, Astana, Kazakhstan; ^4^ Institute of Botany and Phytointroduction, Almaty, Kazakhstan; ^5^ Faculty of Chemistry, Al-Farabi Kazakh National University, Almaty, Kazakhstan; ^6^ Faculty of Geografy, Al-Farabi Kazakh National University, Almaty, Kazakhstan

**Keywords:** *R. linearifolia* Boriss., ecopopulations, metabolome, polymorphism, iPBS amplification profiling, ISSR amplification profiling

## Abstract

*Rhodiola linearifolia* Boriss. (*R. linearifolia*) has long been used in folk medicine; however, research on its resources is limited due to the species inaccessibility for collection, and its potential for cultivation has not been previously explored. Understanding the synthesis of its valuable metabolites, as well as the factors influencing their accumulation under natural conditions, remains a relevant research objective. In this study, the metabolome of various ecopopulations of *R. linearifolia* was analyzed, and the relationship between metabolomic variations and genetic polymorphism was investigated using inter-simple sequence repeat (ISSR) and inter-primer binding site (iPBS) markers. Several significant positive correlations (r = 0.50–0.89) were observed. Significant genetic and metabolomic differences were identified among ecopopulations. Notably, experimental confirmation was obtained showing that ecopopulations with higher genetic polymorphism exhibited a more diverse metabolomic profile. These findings contribute to a deeper understanding of the species’ adaptation mechanisms and provide a foundation both for developing approaches to *in situ* conservation for *R. linearifolia in situ* in natural habitats, and optimizing introduction strategies for cultivation outside its natural range.

## 1 Introduction

The production of pharmaceuticals from plant-derived raw materials in sufficient quantities depends on the availability and sustainability of medicinal plant resources, which are influenced by both climatic factors and the risk of overexploitation of natural populations. In the context of global climate change, it is essential to assess and thoroughly investigate the resource potential of plant species with promising medicinal and veterinary applications, as well as to identify optimal strategies for the cultivation and conservation of rare but valuable species.

The *Crassulaceae* family has long been used in both traditional and official medicine in Kazakhstan and worldwide. However, the resources of many valuable species remain limited due to past unsustainable harvesting practices or their natural inaccessibility, while their potential for cultivation has been largely unexplored. The genus *Rhodiola* L., which includes 9–12 species in Kazakhstan according to different sources (Pavlov, 1956; Baitenov, 2001), is typically found in remote habitats, often growing in rocky soils or crevices within subalpine and alpine zones. This study focuses on *Rhodiola linearifolia* Boriss. (*R. linearifolia*), a rhizomatous perennial characterized by a robust rhizome covered with scaly leaves. The species typically produces 2–6 stems, reaching up to 40 cm in height, with densely arranged linear leaves measuring 2–5 cm in length. Its inflorescence is corymbose and multi-flowered, with a blooming period from June to July. As a psychrophyte, *R. linearifolia* has a short growing season that ends in response to early frosts or the onset of drought conditions, usually between late July and early August. It thrives in forest meadows, rocky landscapes, and along riverbanks at elevations of up to 3000 m above sea level (m a.s.l.) (Ivaschenko, 2008). Due to its exposure to harsh meteorological conditions and challenging growing environments, *R. linearifolia* is considered a difficult-to-access and slow-recovering species. Additionally, the plant’s extremely small seeds struggle to compete with other species, further limiting natural regeneration. Despite its known medicinal properties (Chaldanbayeva, 2006), research on *R. linearifolia* remains limited, and little is known about how environmental conditions influence its physiological traits and bioactive compounds (Terletskaya et al., 2024).

It is crucial to recognize that the current distribution of plants such as *R. linearifolia* results from a combination of environmental conditions, the species’ ecophysiological potential, and its species-specific migratory capacity. The increasing negative impact of environmental factors, along with their duration and intensity, determines the plant’s adaptive stress responses. Scientific literature provides evidence that plants growing under harsh climatic conditions tend to exhibit higher concentrations of secondary metabolites compared to those of the same species cultivated under optimal growth conditions (Futuyma and Agrawal, 2009; Li et al., 2015). From a pharmaceutical perspective, this variability leads to significant fluctuations in the content of bioactive compounds, which depend on collection location, plant phenological stage, timing of collection, and the specific plant organs harvested. Therefore, it is essential to determine not only the presence of valuable metabolites but also the factors influencing their synthesis and accumulation (Caretto et al., 2015; Akhi et al., 2021). Studying the synthesis of plant metabolites in natural conditions provides particularly valuable insights. The analysis of samples collected *in situ* offers a more comprehensive understanding than any stress conditions artificially created in a laboratory, as plants in natural environments are influenced by a complex array of biotic and abiotic factors (Dassanayake et al., 2009). However, due to the multitude of factors governing secondary metabolism in nature, it is unsurprising that current knowledge of plant metabolic structures and the pathways involved in their formation remains incomplete (Fernie and Tohge, 2017; Chen et al., 2021). We hypothesize that plant metabolomes not only differ in response to stressors and between species but are also likely to vary among populations of the same species growing under different environmental conditions.

One of the most effective methods for identifying these compounds is gas chromatography–mass spectrometry (GC–MS). This technique enables the simultaneous isolation and analysis of compounds using a mass detector and readily available GC–MS libraries, providing essential information on the physiological state of plants at different growth stages and in response to various environmental stressors (Harb et al., 2010; Barton and Boege, 2017). GC–MS is widely used in modern biological research for profiling stress-responsive metabolites that contribute to plant adaptation under adverse conditions (Fernie et al., 2004; Obata and Fernie, 2012), and it was employed in this study.

However, the analysis of metabolomic spectra alone is insufficient for developing strategies for the conservation of medicinal plants in natural habitats and for establishing effective introduction approaches. Literature data indicate that secondary metabolites, which increase in concentration under the influence of various stressors, play a signaling role in regulating genes responsible for plant protection (Akhi et al., 2021). To establish population identity and determine intraspecific phylogenetic relationships, genotyping must be conducted. However, unlike the transcriptome and proteome, the metabolome is not always directly correlated with the plant genome (Alseekh et al., 2018). Nevertheless, molecular genetic studies employing various marker systems can reveal additional characteristics that have a stronger functional relationship with metabolite composition. This approach can significantly aid in the selection of suitable genotypes for raw material procurement and species introduction. A fundamental task in botany and genetics is the study of genetic diversity and polymorphism, as it provides insights into evolutionary processes occurring within populations and enables the assessment of their adaptive potential. Genetic diversity can be examined using various molecular markers, which are widely applied in genotyping and population structure analysis. ISSR (Inter Simple Sequence Repeat) markers, for instance, are based on the amplification of DNA regions located between simple sequence repeats (SSRs). This method does not require prior knowledge of the genome sequence and provides a high degree of polymorphism, making it useful for assessing genetic variability, genome mapping, and analyzing phylogenetic relationships at both interspecific and intraspecific levels. The application of ISSR markers allows for precise determination of the genetic structure of populations and the assessment of genetic diversity across a wide range of plant species, including rare and endemic ones (Zietkiewicz et al., 1994; Bornet and Branchard, 2001; Contreras et al., 2020; Cultrera, 2023). Similarly, iPBS (Inter-Primer Binding Site) markers are based on the amplification of DNA regions flanked by retrotransposon primer binding sites. This method enables the assessment of polymorphism based on the presence or absence of retrotransposon elements, which are widespread within plant genomes. iPBS markers are highly reproducible and allow for the identification of unique genetic profiles. They are particularly effective for studying polymorphism in species with genomes containing a substantial number of retrotransposons, which function as key regulators of epigenetic changes under short-term stress conditions and may serve as primary drivers of species evolution (Frost et al., 2005; Kalendar et al., 2010, 2011; Terletskaya et al., 2023b, 2023a).

It is expected that the application of ISSR and iPBS markers will enable a more in-depth analysis of genetic differences between populations, facilitating the identification of optimal growth conditions for *R. linearifolia* and contributing to biodiversity conservation efforts. The findings of this study are anticipated to enhance understanding of the adaptive mechanisms and evolutionary processes occurring within *R. linearifolia* populations. Moreover, these insights will support the development of effective species introduction strategies for cultivation outside its natural range, that preserve its pharmacologically valuable properties while ensuring the long-term conservation of this species *in situ* in natural habitats.

## 2 Materials and methods

### 2.1 Research objects

The study was conducted on *Rhodiola linearifolia* Boriss. (*R. linearifolia*) plants from various ecological and geographical populations, collected from their natural habitats in the Northern Tien Shan Mountain range (Kungey, Trans-Ili, and Ketmen) within the Kegen, Talgar, and Uyghur districts of the Almaty region (**Table 1**).

**Table 1:** Site characteristics of *R. linearifolia* natural populations.

Site	GPS coordinates	Altitude, m a.s.l.	Temperature, °C	Air humidity, %	Location
YT	N 43°01’59.8’’ E 78°14’73.9’’	2 020	15.0	83.3	Almaty region, Kungey Alatau Ridge, Shelek River Floodplain, Teklie Sai
QUR	N 43 00’28.4’’ E 78 18’41.4’’	2 112	16.0	82.2	Almaty region, Kegen District, Kungey Alatau Ridge, Kurmekti Gorge
KET	N 43 24’32.9’’ E 80 30’48.8’’	2 275	15.0	79.7	Almaty region, Uyghur District, Ketmen Ridge, Tetirputak Gorge, 20 km Northeast of Ulken Delkan village
MED	N 43°09’41” E 77°05’42”	2 557	10.0	69.4	Almaty region, Ile-Alatau State Nature Reserve
BAU	N 43 03’86.5’’ E 76 59’26.3’’	2 557	8.6	69.4	Almaty region, Big Almaty Gorge

An analysis of the identified populations of *R. linearifolia* indicates that the species is typically found in a scattered distribution, either as solitary shrubs or in small clusters. The area occupied by these populations ranges from 100 to 2,000 m^2^. Within different plant communities, the proportion of *R. linearifolia* varies between 3% (occasionally up to 5%) and 10% (occasionally up to 15%). On a 100 m^2^ area, an average of 8–10 generative individuals can be found, each producing 3 to 6 shoots, along with approximately 5 young vegetative plants bearing 2 to 3 shoots. Overall, the number of generative individuals within all studied populations remains low, ranging from 3 to 10 specimens per 100 m^2^. The highest abundance of *R. linearifolia* individuals was observed in populations located on the Kungey Alatau Ridge, where ecological conditions appear to be the most favorable for its growth, and anthropogenic impact is minimal.

### 2.2 Metabolomic analysis

The analysis of biologically active organic compounds was carried out using gas chromatography–mass spectrometry (GC-MS) on an Agilent 7890A/5975C system (Agilent Technologies, Santa Clara, CA, USA). Depending on the size (i.e. the number of plants) of each population, between one and seven subpopulations were analyzed per ecopopulation. These were located 10 m apart to prevent the inclusion of vegetatively propagated individuals. Each sample consisted of 3 to 5 shoots collected from separate, individual plants. Аll metabolomic samples were analyzed in triplicate. Plant tissues were preserved in 96% ethanol at a ratio of 100 g to 500 mL and extracted twice over 72 hours each on an orbital shaker until the ethanol became clear and colorless. Each sample (0.7 µL) was injected into the GC-MS at 310°C in splitless mode.

Compound separation was performed on a DB-17MS capillary column (60 m × 0.25 mm × 0.25 µm, Agilent Technologies, Santa Clara, CA, USA) using helium as the carrier gas at a flow rate of 1 mL min^−1^. The oven temperature was programmed to increase from 50 to 300°C at a rate of 5°C min^−1^, with a final hold at 300°C for 10 min. Detection was performed in SCAN mode with an m/z range of 34–800.

System control and data analysis were conducted using Agilent MSD ChemStation software (v. 1701EA, Agilent Technologies, Santa Clara, CA, USA). Retention times, peak areas, and spectral data from the mass spectrometer were analyzed. Compound identification was performed by interpreting mass spectra using the Wiley 7^th^ Edition and NIST 11 libraries, which collectively contain over 550,000 spectra.

To evaluate the reproducibility of the GC-MS-based metabolomic profiling method, the relative standard deviation (RSD%) was calculated based on three independent replicate analyses for each sample. The RSD% values for the main compound classes ranged from 1.7% to 8.8%, which fall within the acceptable threshold (<10%) for GC-MS analyses, thereby confirming the reliability and reproducibility of the analytical procedure.

To process results of metabolomic analysis, average value for each ecopopulation was calculated. All experiments were performed in triplicate.

### 2.3 Genetic analysis

For the genetic analysis of *R. linearifolia* populations using iPBS and ISSR profiling, ten plants were selected, each growing at least 10 m apart to eliminate the possibility of vegetative reproduction.

DNA extraction from *R. linearifolia* was performed using a CTAB-HEPES lysis buffer in the presence of RNase A (2% CTAB, 2 M NaCl, 10 mM Na_3_EDTA, 50 mM HEPES, pH 5.3) (Kalendar et al., 2021). DNA integrity was assessed via electrophoresis on a 1% agarose gel, run in a chamber containing 1× THE buffer (20 mM Tris-HEPES, pH 8.06). Gel imaging was conducted using the iBright CL1500 gel documentation system (Invitrogen). DNA concentration was determined spectrophotometrically using a NanoDrop 1000 spectrophotometer (Thermo Scientific, Madison, USA).

For genetic diversity assessment, universal PBS primers complementary to the PBS regions of various retrotransposons, along with universal ISSR primers, were used. The sequences and characteristics of the primers are provided in **Table 2**.

**Table 2:** iPBS and ISSR primers’ sequences and characteristics.

ID	Sequence	Tm (°C)	CG (%)	Amplicons (bp)
iPBS primers				
2221	ACCTAGCTCACGATGCCA	58.0	55.6	200-3500
2225	AGCATAGCTTTGATACCA	57.0	39.0	200-3000
2226	CGGTGACCTTTGATACCA	61.0	50.0	450-3000
2228	CATTGGCTCTTGATACCA	51.9	44.4	650-2900
2230	TCTAGGCGTCTGATACCA	54.0	50.0	200-6000
2232	AGAGAGGCTCGGATACCA	56.6	55.6	300-5000
2240	AACCTGGCTCAGATGCCA	58.9	55.6	300-5000
2256	GACCTAGCTCTAATACCA	56.0	44.0	200-3200
2397	ATGGTCGCTCTGATACCA	53.0	50.0	850-9000
ISSR primers				
1813	AGCAGCAGCAGCAGCAGCC	72.0	68.4	200-1150
1814	CTCCTCCTCCTCCTCCTCG	66.0	68.4	300-1300
1816	CTCTCTCTCTCTCTCTCTCTG	62.0	52.0	250-1500
1818	ACACACACACACACACACACG	68.0	52.0	100-2000
1819	ACACACACACACACACACACT	67.0	48.0	300-1600
1820	AGAGAGAGAGAGAGAGAGAGC	64.0	52.0	200-1300

Polymerase chain reaction (PCR) amplification was carried out in a 20 μL reaction mixture containing 3 μL of DNA (10 ng μL^−1^), 1 μL of primer (10 mM), and 1 U of Phire Plant Direct PCR Master Mix (Thermo Scientific). The amplification protocol consisted of an initial denaturation at 98°C for 2 min, followed by 30 cycles of denaturation at 98°C for 30 sec, annealing at 50–57°C for 1 min, and extension at 72°C for 1 min. A final elongation step was performed at 72°C for 2 min. Amplifications were conducted using a SimpliAmp thermal cycler (Thermo Fisher Scientific Inc., USA). To ensure reproducibility, each DNA sample was analyzed in duplicate.

Amplification products (amplicons) were visualized on a 1.5% agarose gel containing ethidium bromide. The sizes of the amplified DNA fragments were determined by comparison with a molecular weight marker (Thermo Scientific GeneRuler DNA Ladder Mix, 100–10,000 bp). Fragment lengths were analyzed using the iBright CL1500 gel documentation system software (Thermo Fisher Scientific). The level of polymorphism was calculated as the percentage of polymorphic amplicons relative to the total number of amplicons for each primer.

The gels were analyzed using the fingerprinting method, which involved the creation of a binary matrix in which the presence of a fragment was scored as 1 and its absence as 0. Clustering analysis based on this matrix was performed using the unweighted pair-group method with arithmetic mean (UPGMA) in the Molecular Evolutionary Genetics Analysis (MEGA-X) software. Tajima’s test statistics were calculated in MEGA-X (Kumar et al., 2018).

All experiments—from the selection of effective primers to the genetic analysis—were performed in triplicate for each DNA sample to ensure the reproducibility of the genotyping results. This approach enabled the identification of consistent and sample-specific iPBS profiles.

Key indicators of genetic diversity, including the number of effective alleles, heterozygosity, Shannon’s information index (I), and the genetic differentiation index (PhiPT), were calculated using GenAlex 6.5 (Peakall and Smouse, 2006). Additionally, molecular variance (AMOVA) was assessed both among and within populations using GenAlex 6.5. The resulting dendrogram was constructed using the UPGMA method (Kumar et al., 2018).

## 3 Results

### 3.1 Metabolomic analysis of *R. linearifolia* ecopopulations

**Figure 1:**
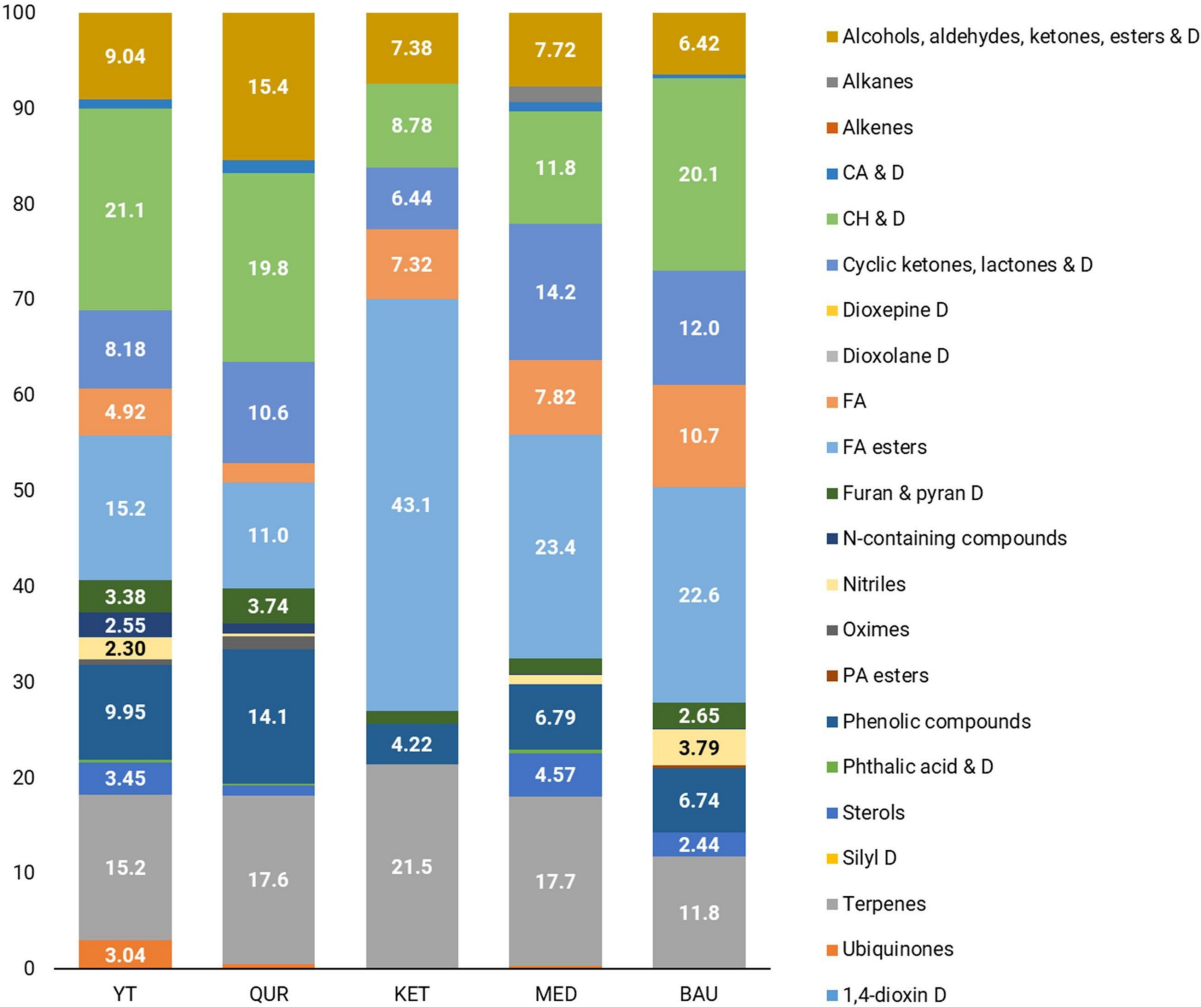
Metabolomic profiles of *R. linearifolia* shoots from different ecopopulations.

**Figure 2:**
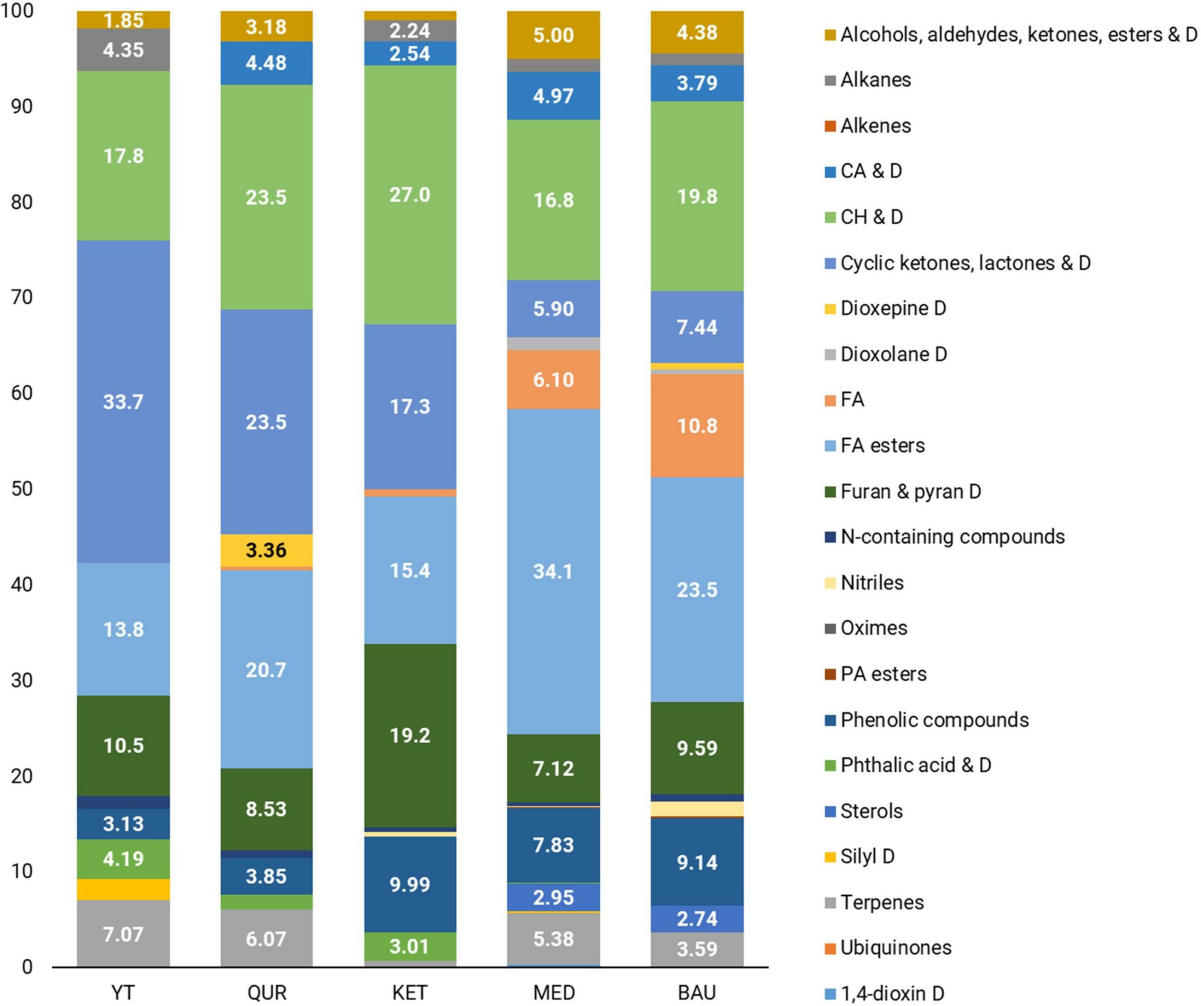
Metabolomic profiles of *R. linearifolia* flowers from different ecopopulations.

GC-MS chromatogram analysis revealed that during the flowering stage, the dominant metabolite classes in *R. linearifolia* shoots include fatty acids (including their ester forms), carbohydrates and their derivatives, terpenes, cyclic compounds (such as ketones, lactones, and their derivatives), phenolic compounds, and a group of alcohol, aldehyde, ketone, and ester derivatives (**Figures 1**, **2**).

In the shoots of *R. linearifolia* plants, metabolite composition varied across ecopopulations. In the KET ecopopulation, fatty acids (including esters) (50.4%) and terpenes (21.5%) were the predominant metabolite classes. In contrast, in the YT, QUR, and BAU ecopopulations, carbohydrates and their derivatives were the most abundant, accounting for 21.1%, 19.8%, and 20.1%, respectively. A relatively high proportion of phenolic derivatives (14.1%) was observed in the shoots of the QUR ecopopulation, while the highest percentage of cyclic compounds (14.2%) was detected in the MED ecopopulation.

In the flowers of *R. linearifolia* during this vegetative stage, the dominant metabolite classes included fatty acids, carbohydrates, phenols, furan and pyran derivatives, terpenes, and phosphoric acid esters. The highest percentage of carbohydrates (27.0%) and furan and pyran derivatives (19.2%) was found in the flowers of the KET ecopopulation. A predominance of fatty acids (including esters) was observed in the MED (40.2%) and BAU (34.3%) ecopopulations. In the YT ecopopulation, cyclic compounds (33.7%) and terpenes (7.07%) were the most abundant, whereas in the QUR ecopopulation, a relatively high content of carbohydrates and their derivatives (23.5%) and cyclic ketones, lactones and their derivatives (23.5%) was noted.

### 3.2 Inter-primer binding site genome fingerprinting analysis of *R. linearifolia* ecopopulations

As shown in **Table 3**, the Na value, which reflects the level of allelic diversity in the studied ecopopulations, ranged from 0.827 to 1.048, with the highest value observed in the BAU population (1.048). Similarly, the Ne indicator (effective number of alleles) was highest in the BAU population (1.266), indicating greater genetic diversity compared to the other ecopopulations. The Shannon index (I) varied between 0.179 and 0.248, with the highest value (0.248) also recorded for the BAU population. The Ne value was consistently higher than the average number of alleles per population, and in the KET population, this value was more than twice as high. This finding may suggest significant population isolation or limited gene flow within the population. Expected heterozygosity (He) ranged from 0.114 to 0.162, with the highest value observed in the BAU population (0.162). Unbiased expected heterozygosity (uHe) varied from 0.127 to 0.180, with the highest value (0.180) also recorded in the BAU population. The number (%) of polymorphic loci (NPB) was consistent with Na values across all populations, further confirming the level of allelic diversity.

**Table 3:** Genetic diversity of *R. linearifolia* ecopopulations based on genetic polymorphism data from ISSR and iPBS profiling.

Population	Na	Ne	I	He	uHe	R	NPB
According to iPBS profiling							
BAU	1.048	1.266	0.248	0.162	0.180	12	50.0
QUR	0.923	1.211	0.205	0.132	0.147	5	42.9
MED	0.827	1.183	0.182	0.116	0.129	10	39.3
YT	0.935	1.182	0.197	0.122	0.136	7	45.8
KET	0.500	1.126	0.114	0.075	0.084	5	22.6
mean	0.845	1.192	0.188	0.120	0.134	8	39.9
According to ISSR genotyping							
BAU	0.805	1.152	0.166	0.103	0.114	3	39.0
QUR	0.585	1.167	0.156	0.103	0.114	4	29.3
MED	0.976	1.180	0.208	0.127	0.141	5	48.8
YT	0.293	1.076	0.069	0.046	0.051	0	12.2
KET	0.634	1.188	0.170	0.113	0.126	2	31.7
mean	0.695	1.157	0.160	0.102	0.113	0.695	34.2

Na, number of alleles; Ne, number of effective alleles per locus; I, Shannon’s information index; He, expected heterozygosity; uHE, unbiased expected heterozygosity; R, rare fragments; NPB, number (%) of polymorphic loci.

Analysis of genetic variation between and within *R. linearifolia* ecopopulations using AMOVA revealed that 27% of the total genetic variation was attributed to differences between populations, while 73% was due to within-population variation. The PhiPT statistic (0.265) indicated a moderate level of genetic differentiation among *R. linearifolia* populations, corroborating previously obtained data. Additionally, the probability value P (rand > data) (0.001) confirmed the statistical reliability of the differences between populations, highlighting the high significance of the results (**Table 4**).

**Table 4:** Analysis of molecular variance (AMOVA) of *R. linearifolia* ecopopulations.

Variability	df	SS	MS	Est. Var.	%	PhiPT	P value
According to iPBS profiling							
Between populations	5	226.333	45.267	5.823	27%	0.265	0.001
Within populations	24	387.600	16.150	16.150	73%		
Total	29	613.933		21.973	100%		
According to ISSR genotyping							
Between populations	5	37.600	7.520	0.794	18%	0.183	0.001
Within populations	24	85.200	3.550	3.550	82%		
Total	29	122.800		4.344	100%		

Df, number of degrees of freedom; SS, sum of squares; MS, mean square; Est. Var, variance; PhiPT, index of genetic differentiation of populations.

As shown in **Table 5**, the number of polymorphic (segregating) sites (168) for the PBS loci reflects a high level of genetic variability within the sample. The proportion of segregating sites is close to one (0.95), indicating a high degree of polymorphism, which is characteristic of the DNA markers used in this study. The nucleotide diversity (π = 0.253) similarly suggests substantial genetic variation within the sample and confirms the successful amplification of the expected PBS region sequences of retrotransposons. Moreover, the nucleotide diversity exceeds the Watterson estimator (π ≥ Θ), which may indicate an excess of polymorphic sites. This pattern could result from stabilizing selection, which maintains individuals with high environmental adaptability within the *R. linearifolia* population. Alternatively, it may reflect a reduction in population size or population fragmentation.

**Table 5:** Tajima’s neutrality test results.

m	S	ps	Θ	π	D
according to iPBS profiling					
25	159	0,95	0.250	0,253	0,049
according to ISSR genotyping					
30	38	0.97	0.245	0.203	-0.634

S, number of amplicons; ps, frequencies of the alleles; Θ, number of silent polymorphisms per amplicon; π, amplicons diversity; D, estimated value of Tajima’s test.

**Figure 3:**
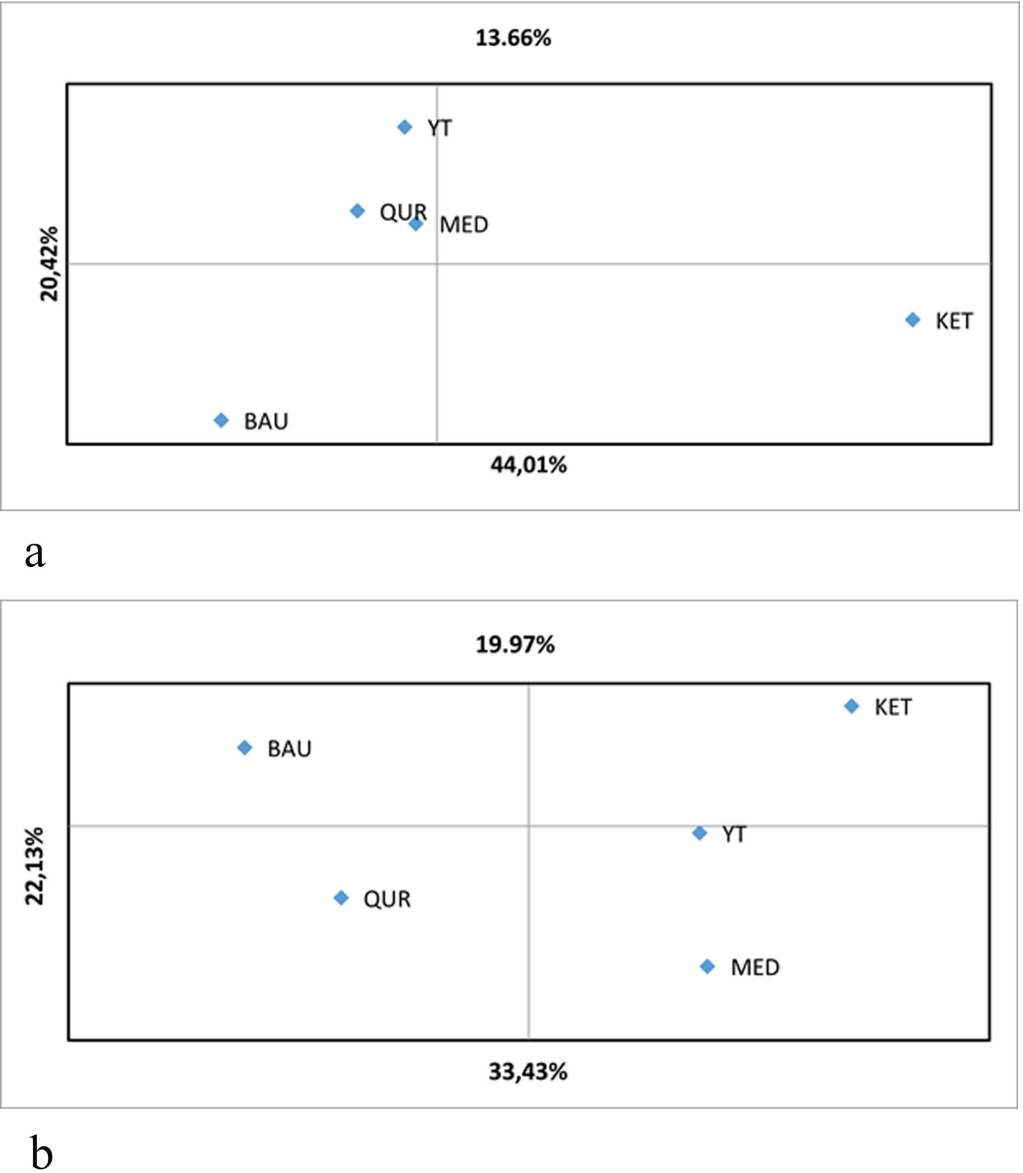
Principal coordinates analysis (PCoA) of *R. linearifolia* ecopopulations based on **(a)** iPBS profiling and **(b)** ISSR genotyping.

The results of the principal coordinates analysis (PCoA) demonstrate genetic differentiation among *R. linearifolia* ecopopulations based on ISSR and iPBS profiling (**Figure 3**). For ISSR analysis, the first principal coordinate explains 33.43% of the total variance, the second accounts for 22.13%, and the third reflects 19.97%, yielding a cumulative explained variance of 75.53%. For iPBS profiling, the first principal coordinate explains 44.01%, the second 20.42%, and the third 13.66%, with a total variance explained of 78.09%.

The first principal coordinate in the ISSR-based PCoA distinctly separates the BAU and QUR populations from the others. In contrast, based on iPBS markers, the KET population is positioned at a considerable distance from the others in the lower right quadrant, indicating substantial genetic divergence. The MED, YT, and QUR populations form a closely grouped cluster, reflecting genetic similarity and moderate differentiation from the BAU and KET populations.

The PCoA plots further illustrate these patterns, with both marker systems distinguishing the BAU and KET populations from the remaining populations along the second principal coordinate. Notably, the first principal coordinate provides the strongest separation between these divergent populations, underscoring their marked genetic differentiation. Overall, the PCoA results reflect both genetic similarity and divergence among populations in relation to their geographic distribution, in agreement with the AMOVA findings.

### 3.3 Genetic polymorphism analysis of *R. linearifolia* ecopopulations using ISSR markers

Analysis of the genetic diversity of *R. linearifolia* populations using ISSR markers provided insights into the genetic polymorphism of the studied ecopopulations (**Table 3**). The results demonstrated varying degrees of genetic heterogeneity. The average number of alleles per locus (Na) ranged from 0.293 in the YT population to 0.976 in MED, indicating differences in allelic diversity. The effective number of alleles (Ne) was highest in the KET population (1.188) and lowest in YT (1.076). The Shannon information index (I), which characterizes genetic variability, varied from 0.069 in YT to 0.208 in MED, indicating different levels of genetic complexity. Observed heterozygosity (He), representing the probability that two randomly selected alleles will be different, also varied among populations, ranging from 0.046 in YT to 0.127 in MED. Unbiased expected heterozygosity (uHe) was lowest in YT (0.051) and highest in MED (0.141). The number of rare alleles (R) ranged from 0 in YT to 5 in MED, reflecting the presence of unique genetic features across ecopopulations.

The percentage of polymorphic loci (NPB) ranged from 12.2% in YT to 48.8% in MED, highlighting significant genetic diversity among the populations, which may be influenced by environmental and geographic factors. Genetic variability analysis revealed that 18% of the total genetic variation was attributed to differences between ecopopulations, indicating moderate genetic differentiation. This finding is supported by the PhiPT index value of 0.183, which signifies statistically significant differentiation among ecopopulations (P = 0.001) (**Table 3**). The majority of genetic variability (82%) was observed within populations, suggesting a high level of genetic diversity at the intrapopulation level. The estimated variance (Est. Var.) between populations was 0.794, while variance within populations was 3.550, emphasizing substantial intrapopulation diversity. The total variance across all populations was 4.344, representing 100% variability.

The Tajima neutrality test (**Table 5**) was performed to evaluate the distribution of allele frequencies across different taxonomic groups and to investigate interpopulation polymorphism at ISSR loci. A slight reduction in nucleotide diversity was observed (π = 0.203), which is lower than the Watterson estimator Θ (0.245), as reflected by the negative Tajima’s D value (-0.634). This may indicate the presence of negative (purifying) selection, as supported by the lower nucleotide diversity index (π ≤ Θ).

### 3.4 Pearson correlation analysis of genetic polymorphisms and metabolomic profiles

**Figure 4:**
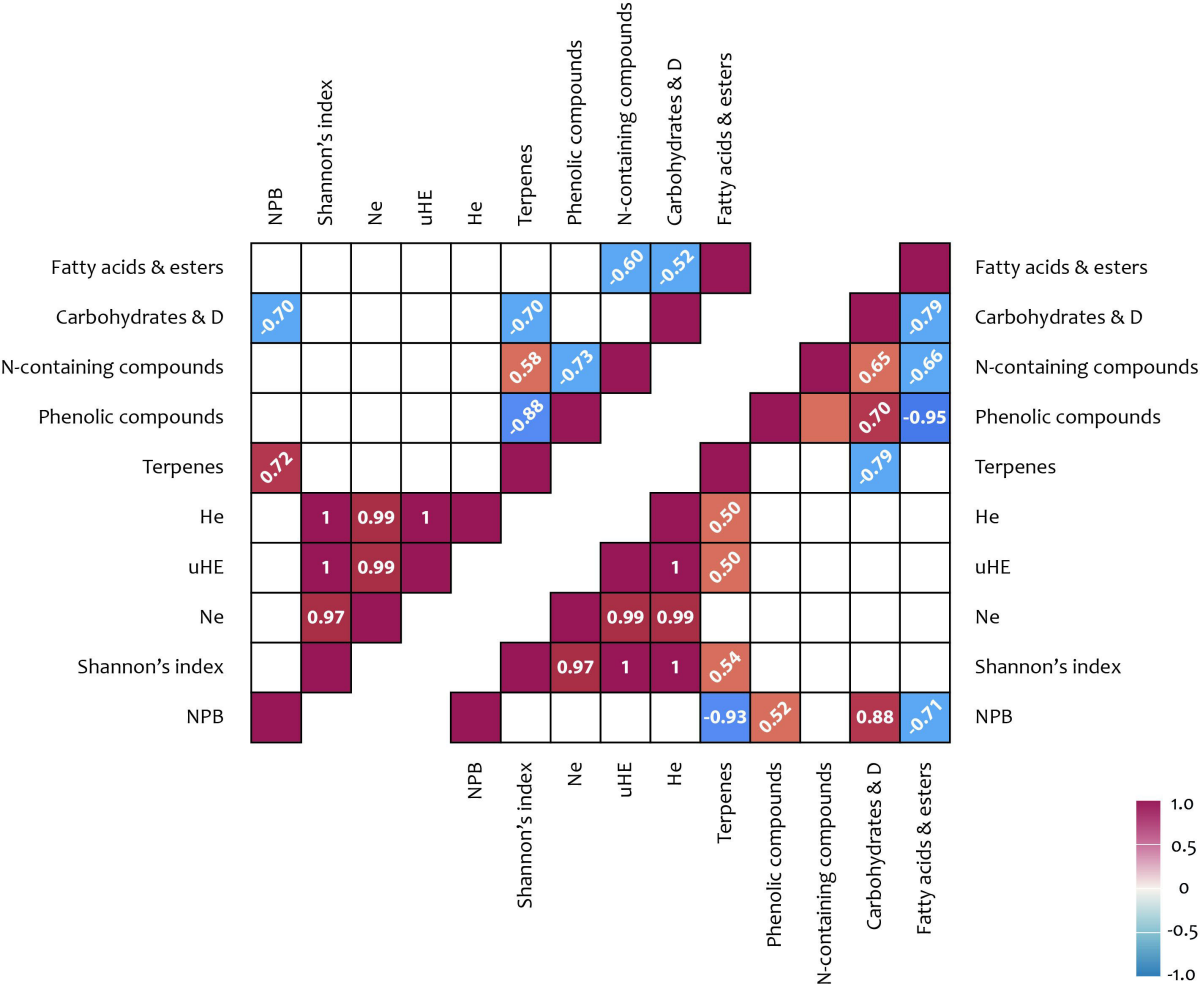
iPBS polymorphism and metabolites correlation results: upper triangle – flowers; lower triangle – shoots. Correlation coefficients (< −0.5 or > 0.5) written at 45° represent not significance but tendencies; coefficient written horizontally are significant (*p* < 0.05); D, derivatives; Na, number of alleles; He, expected heterozygosity; uHE, unbiased expected heterozygosity; NPB, number (%) of polymorphic loci.

Significant positive correlations were observed between carbohydrate content in shoots and the NCB iPBS polymorphism index (r = 0.88), as well as between terpenoid content and multiple iPBS polymorphism indices (r = 0.50–0.54) (**Figure 4**; **Supplementary Figure 1**). In flowers, a positive correlation (r = 0.72) was identified between the NCB iPBS polymorphism index and terpenoid content.

**Figure 5:**
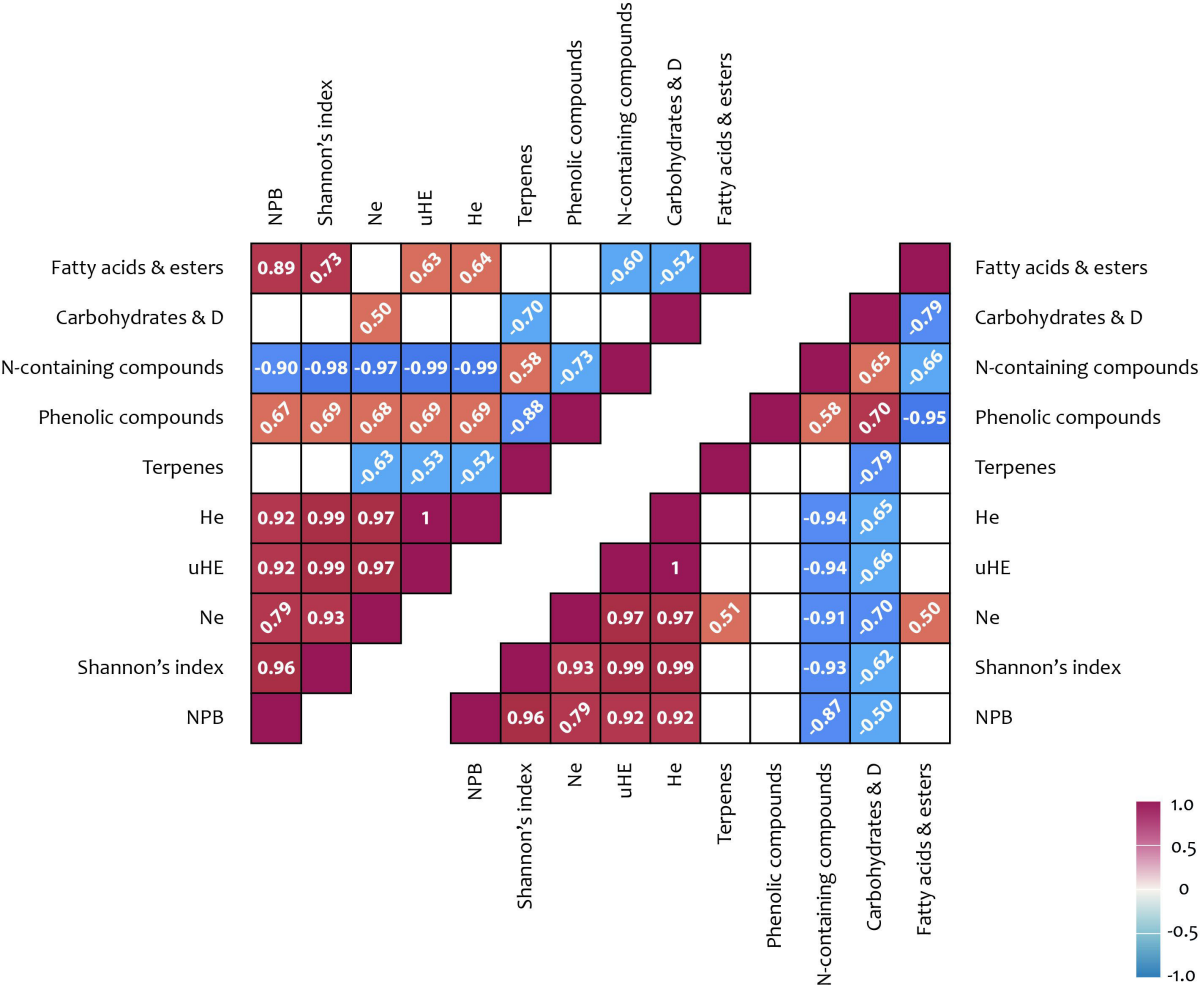
ISSR polymorphism and metabolites correlation results: upper triangle – flowers; lower triangle – shoots. Correlation coefficients (< −0.5 or > 0.5) written at 45° represent not significance but tendencies; coefficient written horizontally are significant (*p* < 0.05); D, derivatives; Na, number of alleles; He, expected heterozygosity; uHE, unbiased expected heterozygosity; NPB, number (%) of polymorphic loci.

**Figure 6:**
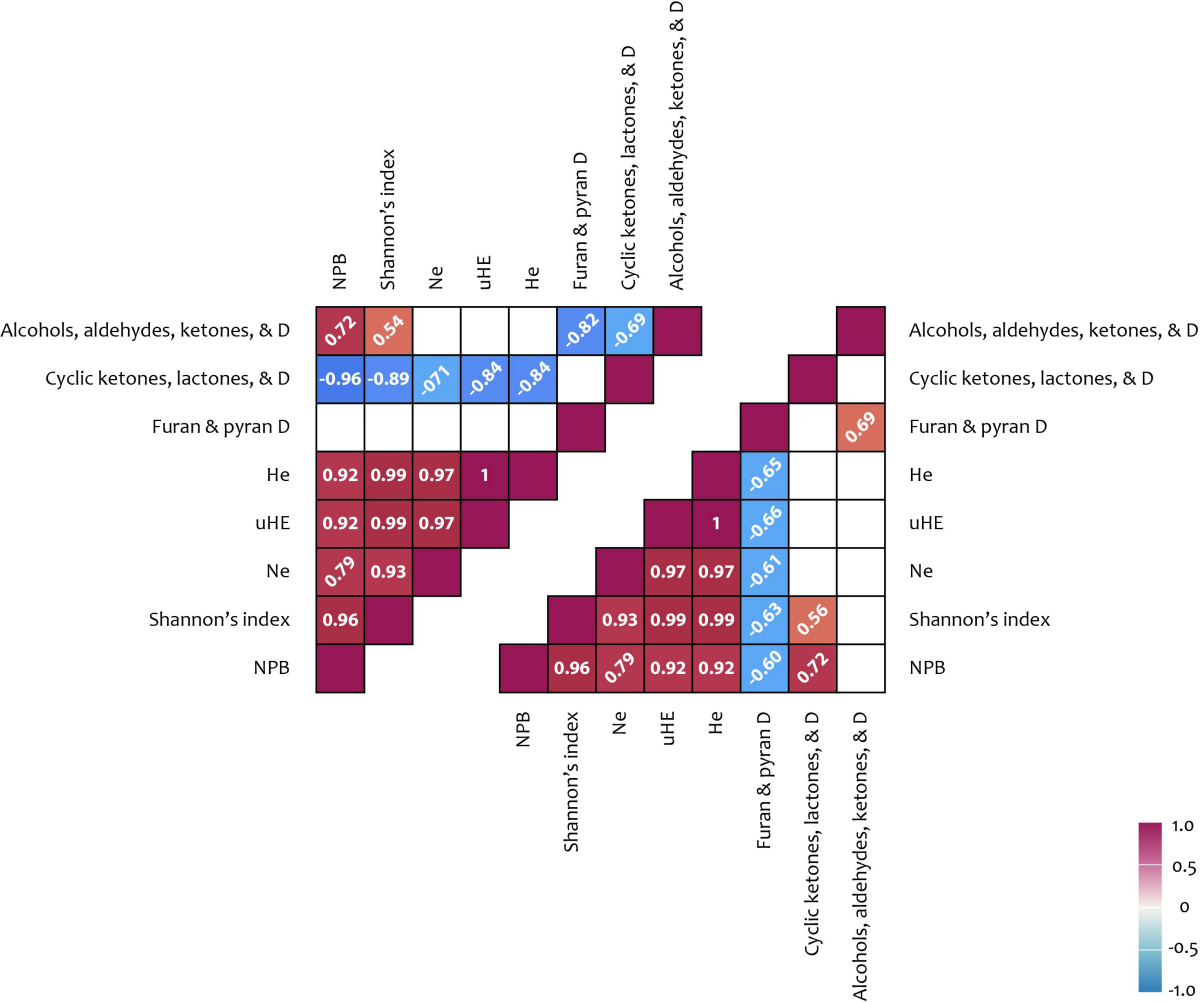
ISSR polymorphism and metabolites correlation results: upper triangle – flowers; lower triangle – shoots. Correlation coefficients (< −0.5 or > 0.5) written at 45° represent not significance but tendencies; coefficient written horizontally are significant (*p* < 0.05); D, derivatives; Na, number of alleles; He, expected heterozygosity; uHE, unbiased expected heterozygosity; NPB, number (%) of polymorphic loci.

An analysis of the potential relationships between metabolite content and ISSR polymorphism indices revealed several significant positive correlations. A correlation was observed between carbohydrate content and the number of effective alleles per locus (Ne) (r = 0.50). Additionally, a positive correlation was found between the number of effective alleles per locus and terpenoid content in shoots (r = 0.51). Further correlations were identified between the percentage of polymorphic loci (NPB), the Shannon index, and the cyclic compound content in shoots (r = 0.72 and 0.51, respectively). Moreover, significant associations were detected between several ISSR polymorphism indices and phenolic compound content (r = 0.67–0.69), as well as between ISSR polymorphism indices and fatty acid content (including derivatives) in flowers (r = 0.63–0.89) (**Figures 5**, **6**).

## 4 Discussion

A population, as an integrated biological system, is always in equilibrium with its surrounding environment. To minimize fluctuations in population size and enhance its integrity, the population must maintain homeostasis, which is supported by various mechanisms—among which polymorphism is one of the most effective [4].

The phytochemical parameters studied in our research vary in response to diverse environmental factors and, at the same time, play a crucial role in signaling responses and acclimatization. However, plant phytochemical characteristics, including metabolite composition and content, are rarely utilized for assessing intraspecific diversity (Lahuta et al., 2018, 2019; Budny et al., 2021; Ibrahim et al., 2021). One of the primary challenges in studying plant metabolism, particularly secondary metabolism, is the widespread occurrence of whole-genome duplication in angiosperms. This duplication has contributed to metabolic diversity over evolutionary time (Kim and Buell, 2015). In this study, a combined genetic and phytochemical approach was employed to characterize different ecopopulations of *R. linearifolia*. It was hypothesized that such a correlative analysis would enable comparisons between the main classes of secondary metabolites and the identified polymorphisms based on retrotransposon and ISSR markers.

The analysis of genetic diversity and polymorphism using PBS markers confirmed that the BAU population exhibited the highest level of genetic diversity across all parameters, indicating its strong genetic potential. In contrast, the genetic diversity of the QUR and YT ecopopulations was more restricted. Furthermore, in the PCoA graph, the KET ecopopulation was positioned at a significant distance from the other populations, highlighting its distinct genetic differentiation. The Tetirputak Gorge of the Ketmen (Ketpen) Range, the habitat of the KET ecopopulation, is a remote and poorly studied region situated within the desert zone. This mountain range spans the territories of two countries—Kazakhstan and China—and is separated from the Central Tien Shan by the high-altitude Kegen Plain, which reaches elevations of up to 2,300 m and can be a potential barrier for the exchange of genetic material. The Ketmen Range is characterized by unique natural resources and hosts a rich flora that differs markedly from that of neighbouring regions. It is notable for the presence of relict plant elements of varying ages, origins, and historical backgrounds (Sadyrova and Baizhigitov, 2019).

The expression of most plant retrotransposons is believed to be activated at specific stages of plant development in actively functioning tissues (Pouteau et al., 1991; Manninen and Schulman, 1993; Kalendar et al., 2004) or in response to abiotic stress factors such as cold, heat, and high solar insolation (Grandbastien, 1998; Takeda et al., 1998; Sormacheva and Blinov, 2011). Many abiotic factors, the intensity of which can fluctuate due to climate change, regulate various biochemical processes, often disrupting the balanced distribution of carbohydrates within plant cells and their transport from source organs to sink organs (Jeandet et al., 2022).

The availability of carbon resources plays a crucial role in determining plant growth patterns, as plants adjust their development accordingly (Marak et al., 2003). Alterations in carbohydrate balance represent a fundamental mechanism that synchronizes internal developmental programs with environmental changes (Stamp, 2003; Hartmann, 2007). It has been suggested that carbohydrate availability influences plant responses to light, soil quality, and various stress conditions (Griffiths et al., 2016). Carbohydrates function similarly to phytohormones by generating physiological signals that activate or suppress plant genes, thereby regulating metabolism (Sheen et al., 1999; Walters and Heil, 2007; Mirajkar et al., 2016). This signaling process enables plants to respond to environmental cues and control resource allocation between tissues and organs (Koch, 1996; Zakhozhiy et al., 2011), ultimately leading to long-term modifications in metabolic activity, resource distribution, and overall plant morphology.

The analysis of carbohydrate profiles in the tissues of the studied ecopopulations revealed significant differences in both the total carbohydrate composition and the relative abundance of individual sugars among *R. linearifolia* ecopopulations. The application of GC-MS enabled the identification of thirteen carbohydrates and their derivatives in stems and eleven in flowers. In this study, the total carbohydrate content in shoots ranged from 8.78% (KET ecopopulation) to 21.1% (YT ecopopulation), while in flowers, it varied from 16.8% (MED) to 27.0% (KET). The predominant carbohydrate in both shoots and flowers was Ethyl α-D-glucopyranoside, with its highest concentration in YT (14.4%) and QUR (14.5%) shoots. Although plants typically store carbohydrates in long polysaccharide chains, sucrose is also a key storage molecule found in plant tissues, including stems, leaves, and flowers. In this study, sucrose was not detected in QUR shoots, while a low concentration (0.61%) was observed in YT shoots. The highest sucrose content in shoots (1.73%) was recorded in the BAU ecopopulation. In flowers, the maximum Ethyl α-D-glucopyranoside content (16.7%) and the highest sucrose content (2.72%) were found in the KET ecopopulation. Sucrose is particularly relevant as it represents the final product of photosynthesis, serves as the primary sugar transported through the phloem, and acts as a carbon skeleton for the synthesis of essential organic compounds, including amino acids and nucleotides (Stein and Granot, 2019). However, it is not the only critical carbohydrate in plants.

A significant negative correlation (r = −0.97, *p* < 0.001) was observed between sucrose content and total soluble carbohydrates in flowers across all studied ecopopulations, whereas no such significant correlation was detected in shoots. Variations in carbohydrate levels among ecopopulations can be explained by the “complete control” model of activity within the source-sink system, which suggests that carbohydrate depletion stimulates photosynthetic enzyme activity in source tissues, while high carbohydrate levels promote accumulation and utilization in sink tissues. Carbon assimilation in plants, which is tightly coordinated with nitrogen uptake, is regulated by the balance of source-to-sink transport processes and is inherently linked to growth conditions (Andersen, 2003; Griffiths et al., 2016; Terletskaya et al., 2022).

The expression of genes involved in carbohydrate balance is regulated by environmental signals, which in turn influence the acclimation potential of plants. Studies have demonstrated that temperature, precipitation, and humidity directly affect plant phenological phases and the accumulation of secondary metabolites, which play a crucial role in adaptation to stressful conditions (Koch, 1996; Zakhozhiy et al., 2011). An analysis of the calculated climate impact index, which considers extreme temperature, precipitation, and relative humidity values during the growing season, was conducted across different climatic zones of Kazakhstan. The findings revealed that high temperatures combined with low precipitation exert the most significant impact on medicinal plants, particularly in arid regions. These climatic conditions promote the enhanced synthesis of bioactive compounds, a phenomenon supported by previous research describing the role of climatic stress in modulating plant metabolism (Koch, 1996). The results underscore the necessity of incorporating climatic factors into conservation and sustainable utilization strategies for rare medicinal species, including *R. linearifolia*.

Although gene-level responses occur more slowly, they determine the magnitude and duration of physiological changes in a way that cannot be achieved by other regulatory mechanisms metabolism (Koch, 1996). The observed correlations between carbohydrate content in shoots and flowers and iPBS polymorphism indices are consistent with previous findings and further emphasize the importance of assessing metabolomic transformations within the source-sink framework. The movement of carbohydrates from source organs to sink organs is essential, as it supplies the necessary substrates for plant reproduction (Ho, 1988; Chang and Zhu, 2017).

During flowering, the primary function of shoots as source organs is to fix CO_2_ through photosynthesis, whereas flowers, as sink organs, largely depend on the organic molecules transported to them (Yang et al., 2002). However, flowers are not merely recipients of carbohydrates and amino acids. In high-altitude plants such as *R. linearifolia*, exposed to intense solar radiation, high temperatures, and low atmospheric CO_2_ concentrations, the biosynthesis of carbon-based compounds such as terpenoids occurs through the combination of five-carbon fragments. These metabolites are essential for growth, development, respiration, and the protection of reproductive organs against various abiotic and biotic stress factors (Rohmer et al., 1996; Borghi et al., 2019; Borghi and Fernie, 2020; Terletskaya et al., 2024). Overall, the findings of this study indicate that the distribution pattern of soluble carbohydrates among ecopopulations, as determined by GC-MS analysis, aligns closely with the patterns revealed by iPBS markers, further supporting the existing literature (Androsiuk et al., 2023).

According to the data presented in **Supplementary Tables 1, 2**, a certain degree of similarity in the metabolomic profiles of the BAU and MED ecopopulations was observed. Extracts from BAU plants contained the highest concentration of fatty acids and a greater diversity of their derivatives, along with the widest variety of carbohydrate derivatives, nitriles, phosphoric acid esters, and furan and pyran derivatives. In contrast, extracts from KET ecopopulation plants exhibited a markedly different metabolomic composition. Notably, sterols were completely absent in both shoots and flowers. Additionally, nitriles, oximes, carboxylic acids and their derivatives, phosphoric acid esters, nitrogen-containing compounds, and ubiquinones were not detected in shoots, which also contained the lowest carbohydrate content (8.78%). Flower extracts from the KET ecopopulation contained minimal amounts of terpenes, with only phytol acetate (0.7%) detected. Similarly, the group of alcohols, aldehydes, ketones, esters, and their derivatives was represented only by benzene-acetaldehyde (0.91%). However, shoot extracts from the KET ecopopulation contained the highest levels of fatty acids and their derivatives (43.4%), as well as terpenes (21.5%). These findings are consistent with **Supplementary Figures 2–8**, which illustrate the similarity in the main metabolite groups between the QUR and YT ecopopulations, as well as the significant differences in both the quantitative and qualitative composition of metabolites in the KET ecopopulation.

Molecular marker technologies employ various approaches based on different principles, yet all contribute to the effective identification of genetic variation loci across the genome (Polat et al., 2015). Among these, ISSR markers are widely utilized for assessing genetic diversity and genetic stability in plants (Wu et al., 2020). In this study, ISSR analysis again confirmed that the BAU population exhibited the highest genetic diversity across all analyzed parameters. In contrast, the KET and MED populations demonstrated lower values for most genetic diversity indicators. However, these populations were found to possess a notable reserve of effective alleles, suggesting genetic isolation, accumulation of unique alleles, and a low level of genetic drift. The results of Principal Coordinates Analysis (PCoA) further reinforced the distinct genetic profile of the KET ecopopulation, which was positioned at a significant distance from the other populations, highlighting its relative uniqueness.

The Tajima neutrality test was conducted to assess allele frequency distributions across taxonomic groups and to evaluate interpopulation polymorphism at ISSR loci (Jarvis et al., 2017; Terletskaya et al., 2023b). A negative Tajima’s D value suggests potential selective pressure or a recent demographic event that may have reduced polymorphism levels. In our case, this may suggest, that is the action of negative (purifying) selection, reducing genetic diversity at the studied loci. Such a process can lead to an increased proportion of individuals with high adaptive potential, particularly following a population bottleneck, potentially facilitating further range expansion.

Additionally, the results of the Tajima test may also reflect underlying demographic dynamics in *R. linearifolia* populations. According to several studies, negative Tajima’s D values may be attributed not only to directional selection acting on genetic markers, but also to reductions in population size (Choudhury et al., 2019; Pot et al., 2005; Tamboli et al., 2016; Zhang et al., 2014). Furthermore, natural selection is directional in nature and often targets functionally significant genetic markers, including iPBS elements, as previously demonstrated for Rhodiola (Terletskaya et al., 2023b). Accordingly, we believe that the negative values observed in the Tajima test reflect a reduction in the number of individuals within *R. linearifolia* populations.

A significant positive correlation was identified between carbohydrate content and the number of effective alleles per locus (Ne) of ISSR polymorphism (**Figures 5**, **6**). Additionally, positive correlations were observed between the number of effective alleles per locus and terpenoid content in shoots, as well as between the percentage of polymorphic loci and the content of cyclic compounds in shoots. Further correlations were found between ISSR polymorphism indices and the content of phenolic compounds and fatty acids (including derivatives) in flowers. Terpenoids in the shoots of high-altitude plants are known to function as antioxidant stress protectors. Terpene hydrocarbons, such as phytols, are integral components of chlorophyll, thereby supporting photosynthetic activity (Farmer, 2001; Sharkey and Yeh, 2001; Boncan et al., 2020; Terletskaya et al., 2021). In addition to their physiological roles, terpenoids also possess notable medicinal properties. Phytol, which was detected in relatively high concentrations (11.0–14.2%) in the shoots of plants across all studied ecopopulations, is a precursor of vitamins E and K1. Lupeol, identified in shoot extracts from the MED, QUR, and KET ecopopulations, and squalene, detected in extracts from the MED and YT ecopopulations, are known for their onco-protective properties. Furthermore, α-amyrin, found in MED, QUR, and KET shoot extracts, exhibits antinociceptive and anti-inflammatory effects. The presence of cyclic compounds (ketones, lactones, and their derivatives) in plant extracts suggests that dynamic transformations within the plant metabolome continuously occur in response to changing environmental conditions (De Jersey and Zerner, 1969; Yunesi, 2005).

Extensive literature highlights the importance of phenolic compounds in photoprotection and the regulation of antioxidant capacity in high-altitude plants (Camas et al., 2014; Padilla-González et al., 2017; Hashim et al., 2020; Nasri et al., 2022). In flower extracts from the BAU and KET ecopopulations, the highest concentrations of phenolic compounds were recorded, reaching 9.14% and 9.99%, respectively. Fatty acids play crucial roles in plant cell membranes, serving as structural components, energy reserves, extracellular barrier storage molecules, and precursors of bioactive compounds involved in stress signaling (Lee et al., 2016; Boncan et al., 2020; Yuldasheva et al., 2020). The highest concentrations of fatty acids (including derivatives) were found in flower extracts from the BAU (34.3%) and MED (40.2%) ecopopulations. According to population genetics principles, plants in natural populations undergo long-term adaptation to specific habitats (Zhuchenko, 2004). The stability of species within populations is essential for maintaining ecological balance and is primarily sustained through high genetic diversity. The findings of this study reveal moderate genetic diversity among the studied *R. linearifolia* ecopopulations, with a complex genetic structure influenced by genetic exchange, local adaptations, and spatial isolation. Metabolome analysis further confirmed a broader adaptive spectrum in the BAU ecopopulation, which exhibited higher genetic polymorphism. Additionally, significant differences were observed in plant extracts from the KET ecopopulation, which also demonstrated notable genetic divergence from the other populations. These findings suggest that *R. linearifolia* employs a diverse array of genetic and phytochemical adaptation mechanisms to cope with varying growth and developmental conditions across ecopopulations.

## 5 Conclusions

The findings of this study reveal both metabolomic and genetic differences among ecopopulations. Experimental confirmation was obtained demonstrating a more diverse metabolomic spectrum in the BAU ecopopulation, which exhibited greater genetic polymorphism. Additionally, significant differences were observed in the metabolomic profile of plants from the KET ecopopulation, which displayed notable genetic divergence from the other populations. These results provide valuable insights into the adaptation mechanisms of *R. linearifolia* and contribute to the selection of optimal strategies for species introduction, as well as the development of conservation approaches for maintaining this species in natural habitats.

## Data Availability

All relevant data is contained within the article: The original contributions presented in the study are included in the article/**Supplementary Material**, further inquiries can be directed to the corresponding author.
