# Supplementary material for: Metabolomic adaptations and genetic polymorphism in ecopopulations of Rhodiola linearifolia Boriss
Source: Front Plant Sci. 2025 Jun 19;16:1570411. doi: 10.3389/fpls.2025.1570411 (PMC12222203; doi:10.3389/fpls.2025.1570411)
Supplement: Supplementary file 1 [file DataSheet1.docx]

Supplementary Material


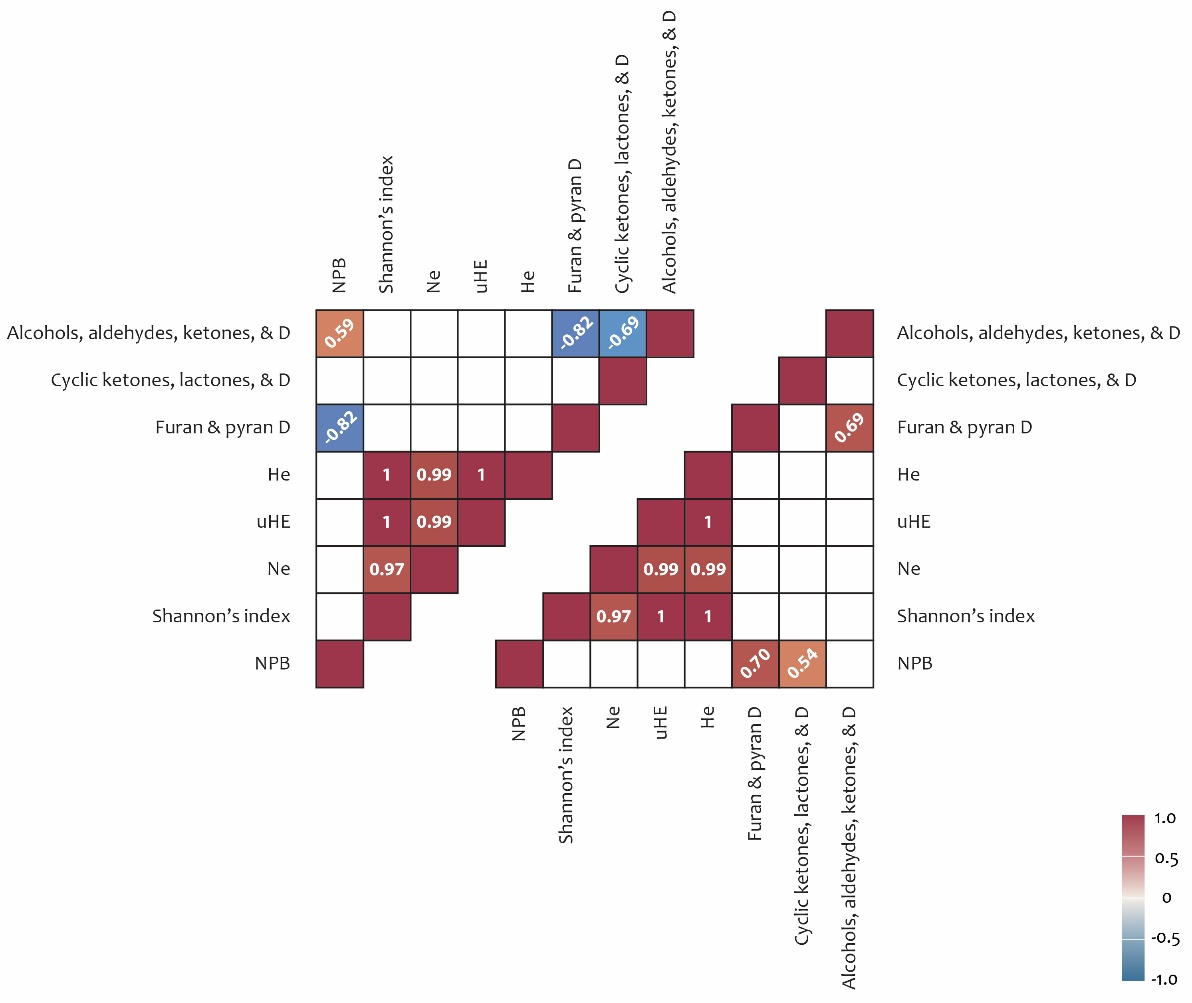


**Supplementary Figure 1.** iPBS polymorphism and metabolites correlation results: upper triangle – flowers; lower triangle – shoots. Notes: correlation coefficients (< -0.5 or > 0.5) written at 45º represent not significance but tendencies; coefficient written horizontally are significant (*p* < 0.05); D – derivatives; Na − number of alleles; He − expected heterozygosity; uHE − unbiased expected heterozygosity; NPB − number (%) of polymorphic loci.

**Supplementary Table 1**. Variation in secondary metabolite content in *R. linearifolia* shoots at different altitudes.

| **RT, min** | **Metabolites** | **MED** | | **YT** | | **KET** | | **QUR** | | **BAU** | |
| --- | --- | --- | --- | --- | --- | --- | --- | --- | --- | --- | --- |
|  |  | Content, % | IP, % | Content, % | IP, % | Content, % | IP, % | Content, % | IP, % | Content, % | IP, % |
| **FATTY ACIDS** | | | | | | | | | | | |
| 33.672 | Hexadecanoic acid | 3,45 | 87 | 0.73 | 70 | - | - | 0.49 | 71 | 4.00 | 74 |
| 37.878 | 9,12-Octadecadienoic acid (Z,Z)- | 1,50 | 85 | 0.90 | 80 | 1.83 | 88 | 0.20 | 89 | 2.65 | 65 |
| 38.428 | 9,12,15-Octadecatrienoic acid, (Z,Z,Z)- | 2,87 | 90 | 3.29 | 77 | 5.48 | 91 | 1.35 | 93 | 4.00 | 86 |
|  | **SUM** | **7.82** |  | **4.92** |  | **7.31** |  | **2.04** |  | **10.7** |  |
| **FATTY ACID ESTERS** | | | | | | | | | | | |
| 7.560 | Propanoic acid, 2-oxo-, methyl ester | 6.12 | 81 | 1.40 | 70 | 2.78 | 70 | - | - | 4.71 | 80 |
| 7.932 | Propanoic acid, 3-(acetylthio)-2-methyl-, (S)- | - | - | - | - | - | - | 0.93 | 69 | - | - |
| 29.734 | Tetradecanoic acid, ethyl ester | 0.09 | 75 | - | - | 0.84 | 85 | 0.30 | 68 | - | - |
| 33.714 | Hexadecanoic acid, ethyl ester | 3.00 | 90 | 1.44 | 90 | 9.23 | 91 | - | - | 1.83 | 89 |
| 34.083 | Ethyl 9-hexadecenoate | - | - | - | - | 0.46 | 75 | - | - | - | - |
| 37.356 | Octadecanoic acid, ethyl ester | 0.40 | 72 | - | - | - | - | - | - | 0.06 | - |
| 37.407 | Ethyl Oleate | 0.14 | 85 | 0.16 | 85 | - | - | - | - | - | - |
| 37.745 | Linoleic acid ethyl ester | - | - | - | - | - | - | 0.19 | 82- | - | - |
| 37.748 | 9,12-Octadecadienoic acid, ethyl ester | 1.97 | 86 | 1.66 | 90 | 4.99 | 92 | 1.22 | 89 | 2.28 | 91 |
| 38.272 | Ethyl 9,12,15-octadecatrienoate | 3.52 | 93 | - |  | - |  | - |  | 0.81 | 93 |
| 38.284 | 9,12,15-Octadecatrienoic acid, ethyl ester, (*Z,Z,Z*)- | 1.56 | 93 | 5.76 | 94 | 14.56 | 96 | 8.36 | 94 | 3.70 | 94 |
| 40.761 | Hexadecanoic acid, 1-(hydroxymethyl)-1,2-ethanediyl ester | - | - | - | - | - | - | - | - | 0.05 | 69 |
| 45.169 | Hexadecanoic acid, 2-hydroxy-1-(hydroxymethyl)ethyl ester | 3.19 | 88 | 1.35 | 80 | 2.05 | 80 | - | - | 3.08 | 80 |
| 48.654 | 9,12-Octadecadienoic acid (Z,Z)-, 2-hydroxy-1-(hydroxymethyl)ethyl ester | 0.37 | 61 | - | - | - | - | - | - | 2.86 | 75 |
| 48.659 | 9,12,15-Octadecatrienoic acid, 2,3-dihydroxypropyl ester, (Z,Z,Z)- | - |  | - | - | - |  | - | - | 0.29 | 81 |
| 48.66 | 9,12-Octadecadienoic acid (Z,Z)-, 2,3-dihydroxypropyl ester | 0.67 | 66 | 1.23 | 75 | 2.89 | 81 | - | - | - | - |
| 49.208 | 9,12,15-Octadecatrienoic acid, 2-phenyl-1,3-dioxan-5-yl ester | - | - | - | - | - | - | - | - | 0.36 | 63 |
| 49.209 | Linolenic acid, 2-hydroxy-1-(hydroxymethyl)ethyl ester (Z,Z,Z)- | 2.40 | 71 | - | - | - | - | - | - | 2.04 | 75 |
| 49.216 | Butyl 9,12,15-octadecatrienoate | - | - | 2.15 | 79 | 5.28 | 82 | - | - | 0.51 |  |
|  | **SUM** | **23.4** |  | **15.2** |  | **43.1** |  | **11.0** |  | **22.6** |  |
| **CARBOHYDRATES AND THEIR DERIVATIVES** | | | | | | | | | | | |
| 21.211 | 1,4:3,6-Dianhydro-α-d-glucopyranose | - | - | 0.20 | 74 | - | - | 0.57 | 69 | - | - |
| 26.496 | α-D-Lyxofuranoside, methyl | 0.31 | 94 | - | - | - | - | 0.45 | 70 | - | - |
| 26.504 | β-D-Lyxofuranoside, methyl | - | - | 1.01 | 72 | 1.24 | 73 | 1.10 | 73 | 0.10 | 75 |
| 28.051 | Sucrose | 1.45 | 72 | 0.61 | 70 | - | - | - | - | 1.73 | 71 |
| 29.615 | *β*-*D*-Glucopyranose, 1,6-anhydro- | 1.61 | 87 | 2.06 | 83 | - | - | 1.37 | 84 | 0.71 | 86 |
| 29.633 | D-Allose | 0.32 | 86 | - | - | 1.35 | 85 | 1.82 | 83 | 0.29 | 89 |
| 30.627 | β-D-Glucopyranoside, methyl | 0.21 | 69 | - | - | - | - | - | - | 0.31 | 64 |
| 31.518 | Ethyl *α*-*D*-glucopyranoside | 5.81 | 83 | 14.43 | 89 | 6.19 | 83 | 14.48 | 85 | 7.99 | 89 |
| 33.599 | α-D-Galactopyranoside, methyl | 0.14 | 76 | - | - | - | - | - | - | - | - |
| 33.604 | α-D-Glucopyranoside, methyl | - | - | - | - | - | - | - | - | 2.19 | 72 |
| 39.114 | β-D-Glucopyranose, 4-O-β-D-galactopyranosyl- | 0.11 | 63 | - | - | - | - | - | - | 0.16 | 66 |
| 42.686 | α-D-Glucopyranose, 4-O-β-D-galactopyranosyl- | - | - | - | - | - | - | - | - | 2.60 | 70 |
| 42.692 | α-Methyl-D-mannopyranoside | 1.82 | 69 | 2.74 | 69 | - | - | - | - | 4.01 | 68 |
|  | **SUM** | **11.8** |  | **21.1** |  | **8.78** |  | **19.8** |  | **20.1** |  |
| **ALKANES** | | | | | | | | | | | |
| 12.714 | Dodecane | 0.80 | 82 | - | - | - | - | - | - | - | - |
| 18.081 | Tetradecane | 0.73 | 89 | - | - | - | - | - | - | - | - |
| 23.100 | Hexadecane | 0.06 | 73 | - | - | - | - | - | - | - | - |
|  | **SUM** | **1.59** |  | **0** |  | **0** |  | **0** |  | **0** |  |
| **NITRILES** | | | | | | | | | | | |
| 6.913 | 2-Methyl-2-butenenitrile | - | - | - | - | - | - | - | - | 0.50 | 70 |
| 6.916 | 2-Pentenenitrile | 0.46 | 63 | - | - | - | - | - | - | 1.07 | 74 |
| 6.917 | 3-Butenenitrile, 2-methyl- | 0.49 | 65 | 2.30 | 86 | - | - | - | - | 2.22 | 79 |
| 10.891 | Propanenitrile, 3-methoxy- | - | - | - | - | - | - | 0.31 | 71 | - | - |
|  | **SUM** | **0.95** |  | **2.30** |  | **0** |  | **0.31** |  | **3.79** |  |
| **OXIMES** | | | | | | | | | | | |
| 7.495 | Oxime-, methoxy-phenyl- | - | - | 0.57 | 79 | - | - | 1.34 | 80 | - | - |
|  | **SUM** | **0** |  | **0.57** |  | **0** |  | **1.34** |  | **0** |  |
| **ALCOHOLS, ALDEHYDES, KETONES, ESTERS AND THEIR DERIVATIVES** | | | | | | | | | | | |
| 6.475 | (S)-(+)-1,2-Propanediol | - | - | 0.26 | 80 | - | - | - | - | - | - |
| 6.476 | Propylene Glycol | - | - | - | - | - | - | 0.41 | 86 | - | - |
| 6.539 | R-(-)-1,2-propanediol | - | - | - | - | - | - | 0.06 | 84 | - | - |
| 6.667 | Glycolaldehyde dimethyl acetal | 0.17 | 66 | - | - | - | - | - | - | - | - |
| 6.706 | Propane, 1,1-dimethoxy-- | 0,74 | 70 | - | - | 2.50 | 70 | - | - | - | - |
| 7.099 | Diethoxymethyl acetate | - | - | - | - | - | - | 0.63 | 66 | - | - |
| 7.290 | 1,2-Ethanediol, monoacetate | - | - | - | - | - | - | 1.74 | 82 | - | - |
| 7.812 | 3-Butene-1,2-diol | - | - | - | - | - | - | 1.24 | 71 | - | - |
| 9.323 | 2-Propanone, 1-(acetyloxy)- | 0.07 | 81 | - | - | - | - | 0.33 | 84 | 0.08 | 73 |
| 9.325 | 1,2-Ethanediol, diacetate | 0.14 | 67 | - | - | - | - | - | - | - | - |
| 23.185 | 1,2-Ethanediol, 1-phenyl- | - | - | - | - | - | - | 0.09 | 83 | - | - |
| 18.227 | 1,4-Butanediol | - | - | 0.76 | 67 | - | - | - | - | - | - |
| 13.357 | 2-Butanol, 3,3'-oxybis- | - | - | - | - | - | - | 0.13 | 73 | - | - |
| 12.073 | Ethanol, 2,2'-oxybis- | - | - | 0.35 | 78 | - | - | 0.75 | 84 | - | - |
| 28.753 | Ethanol, 2-(dodecyloxy)- | - | - | - | - | - | - | 0.54 | 76 | - | - |
| 22.717 | 1-Undecanol | - | - | - | - | - | - | 0.06 | 82 | - | - |
| 22.717 | 1-Dodecanol | - | - | - | - | - | - | 0.14 | 72 | - | - |
| 12.612 | Monoethanolamine | - | - | - | - | - | - | 0.68 | 70 | - | - |
| 12.545 | Glycerin | 2.77 | 79 | 5.04 | 77 | 2.20 | 75 | 3.36 | 80 | 3.36 | 86 |
| 13.282 | 1-Propanol, 2,2'-oxybis- | - | - | - | - | - | - | 0.06 | 81 | - | - |
| 13.366 | 1-Propanol, 2-(2-hydroxypropoxy)- | - | - | - | - | - | - | 0.25 | 81 | - | - |
| 14.694 | Benzeneacetaldehyde | 0.12 | 82 | 0.60 | 91 | - | - | 0.16 | 65 | - | - |
| 15.121 | 2-Propanone, 1-cyclopropyl- | - | - | - | - | - | - | 0.11 | 69 | - | - |
| 18.204 | Cyclopropyl carbinol | 0.84 | 75 | - | - | 0.68 | 72 | 1.46 | 71 | 1.09 | 74 |
| 18.206 | Pentanal | 0.64 | 80 | 0.36 | 72 | - | - | - | - | 0.36 | 75 |
| 20.435 | Triethylene glycol | - | - | 0.44 | 84 | - | - | 0.84 | 85 | - | - |
| 28.325 | Hexaethylene glycol | - | - | - | - | - | - | 0.29 | 76 | - | - |
| 28.328 | Tetraethylene glycol | - | - | 0.46 | 76 | - | - | 0.57 | 71 | - | - |
| 35.315 | Diethylene glycol monododecyl ether | - | - | - | - | - | - | 0.57 | 83 | - | - |
| 25.207 | 1,3-Dioxolane, 2-ethyl- | 0.20 | 68 | - | - | - | - | - | - | 0.30 | 66 |
| 14.395 | 1,3-Dioxol-2-one,4,5-dimethyl- | 2.03 | 73 | 0.77 | 72 | 2.00 | 70 | 0.96 | 74 | 1.23 | 74 |
|  | **SUM** | **7.72** |  | **9.04** |  | **7.38** |  | **15.4** |  | **6.42** |  |
| **CYCLIC KETONES, LACTONES AND THEIR DERIVATIVES** | | | | | | | | | | | |
| 10.457 | 4-Cyclopentene-1,3-dione | 0.75 | 84 | - |  | - |  |  |  | 0.15 | 77 |
| 11.033 | 1,2-Cyclopentanedione | 1.99 | 92 | 2.22 | 85 | 1.20 | 90 | 1.32 | 94 | 2.10 | 90 |
| 11.195 | 2-Cyclopenten-1-one, 2-hydroxy- | 0.87 | 90 | - | - | - |  | 1.69 | 92 | 0.26 | 89 |
| 13.848 | 1,2-Cyclopentanedione, 3-methyl- | 0.70 | 69 | - | - | - | - | 0.23 | 83 | 0.41 | 83 |
| 13.851 | 2-Cyclopenten-1-one, 2-hydroxy-3-methyl- | 1.29 | 82 | 1.44 | 80 | 1.26 | 82 | 1.40 | 78 | 1.11 | 86 |
| 14.916 | 2-Hydroxy-gamma-butyrolactone | 7.31 | 88 | 3.87 | 88 | 3.53 | 87 | 5.26 | 89 | 5.64 | 89 |
| 15.066 | Cyclopentane, 1-acetyl-1,2-epoxy- | 0.33 | 65 | - | - | - | - | - | - | 0.58 | 66 |
| 16.331 | 2-Cyclopenten-1-one, 3-ethyl-2-hydroxy- | - | - | - | - | - | - | 0.14 | 71 | - | - |
| 21.622 | (*S*)-(+)-2',3'-Dideoxyribonolactone | 1.00 | 89 | 0.65 | 78 | 0.45 | 84 | 0.45 | 88 | 0.44 | 73 |
| 25.559 | 4-(2,6,6-Trimethylcyclohexa-1,3-dienyl)but-3-en-2-one | - | - | - | - | - | - | 0.08 | 75 | - |  |
| 32.188 | α,β-Gluco-octonic acid lactone | - | - | - | - | - |  | - | - | 1.29 | 63 |
|  | **SUM** | **14.2** |  | **8.18** |  | **6.44** |  | **10.6** |  | **12.0** |  |
| **CARBOXYLIC ACID AND THEIR DERIVATIVES** | | | | | | | | | | | |
| 7.359 | Acetic acid, (acetyloxy)- | 0.46 | 67 | 0.78 | -77 | - | - | - | - |  |  |
| 12.658 | Butanoic acid, 4-hydroxy- | - | - | - | - | - | - | 0.89 | 80 | - | - |
| 15.482 | Methyl acetoxyacetate | 0.35 | 70 | - | - | - | - | 0.13 | 68 | 0.35 | 73 |
| 21.058 | Acetic acid, pentyl ester | - | - | 0.224 | 65 | - | - | - | - | - | - |
| 21.059 | [1,1'-Bicyclopropyl]-2-octanoic acid, 2'-hexyl-, methyl ester | - | - | - | - | - | - | 0.17 | 70 | - | - |
| 27.018 | 2-Propenoic acid, 2-methyl-, hexyl ester | - | - | - | - | - | - | - | - | 0.06 | 65 |
| 34.581 | Benzeneacetic acid, 4-hydroxy-3-methoxy-, methyl ester | 0.14 | 66 | - | - | - | - | 0.12 | 70 | - | - |
|  | **SUM** | **0.95** |  | **1.00** |  | **0** |  | **1.31** |  | **0.41** |  |
| **PHOSPHORIC ACID ESTERS** | | | | | | | | | | | |
| 26.318 | Phosphoric acid, diethyl dodecyl ester | 0.04 | 74 | - | - | - | - | - | - | 0.07 | 73 |
| 26.318 | Phosphoric acid, diethyl nonyl ester | - | - | - | - | - | - | - | - | 0.08 | 71 |
| 26.319 | Phosphoric acid, diethyl octyl ester | - | - | - | - | - | - | - | - | 0.12 | 72 |
|  | **SUM** | **0.04** |  | **0** |  | **0** |  | **0** |  | **0.27** |  |
| **FURAN AND PYRAN DERIVATIVES** | | | | | | | | | | | |
| 8.740 | 2-Furanmethanol | - |  | - |  | - |  | 0.17 | 70 |  |  |
| 12.688 | Tetrahydro-4H-pyran-4-ol | - | - | - | - | - | - | - | - | 0.20 | 66 |
| 12.697 | 2(5H)-Furanone | 0.56 | 64 | - | - | - | - | 1.63 | 84 | 1.54 | 65 |
| 12.705 | 2(3H)-Furanone | - | - | 1.49 | 65 | 1.31 | 65 | - | - | - | - |
| 13.107 | Ethyl 2-(5-methyl-5-vinyltetrahydrofuran-2-yl)propan-2-yl carbonate | - | - | 0.54 | 87 | - | - | - | - | - | - |
| 16.359 | 2H-Pyran-3-ol, 6-ethenyltetrahydro-2,2,6-trimethyl- | - | - | 0.59 | 80 | - | - | - | - | - | - |
| 17.425 | 4H-Pyran-4-one, 2,3-dihydro-3,5-dihydroxy-6-methyl- | - | - | - | - | - | - | 0.28 | 75 | 0.04 | 65 |
| 19.333 | Benzofuran, 2,3-dihydro- | 0.61 | 93 | 0.76 | 77 | - | - | 0.99 | 84 | 0.35 | 79 |
| 19.549 | 2(3H)-Furanone, 5-acetyldihydro- | 0.18 | 85 | - |  | - | - | 0.23 | 84 | 0.10 | 83 |
| 19.824 | 4H-Pyran-4-one, 3-hydroxy-2,6-dimethyl- | - | - | - | - | - | - | 0.06 | 68 | - | - |
| 21.614 | 5-Hydroxymethyldihydrofuran-2-one | 0.08 | 79 | - | - | - | - | 0.14 | 78 | 0.12 | 76 |
| 21.621 | 5-Oxotetrahydrofuran-2-carboxylic acid, ethyl ester | - | - | - | - | - | - | 0.24 | 79 | - | - |
| 27.014 | 1,6-Anhydro-2,3-dideoxy-β-D-threo-hexopyranose | 0.05 | 65 | - | - | - | - | - | - | 0.28 |  |
| 29.725 | 5-(1,2-Dihydroxyethyl)dihydrofuran-2-one | 0.10 | 68 | - | - | - | - | - | - | 0.02 | 70 |
|  | **SUM** | **1.58** |  | **3.38** |  | **1.31** |  | **3.74** |  | **2.65** |  |
| **PHENOLIC COMPOUNDS** | | | | | | | | | | | |
| 11.815 | Phenol | - | - | 0.19 | 76 | - |  | 1.21 | 84 | - | - |
| 14.514 | Phenol, 3-methyl- | - | - | - | - | - | - | 0.13 | 65 | - | - |
| 17.215 | Phenol, 4-ethyl- | - | - | - | - | - | - | 0.15 | 64 | - | - |
| 18.799 | Catechol | 2.25 | 89 | 2.90 | 81 | 1.76 | 92 | 2.52 | 89 | 1.55 | 91 |
| 20.698 | 1,2-Benzenediol, 3-methyl- | 0.80 | 80 | 1.10 | 82 | 0.85 | 87 | 1.42 | 76 | 0.27 | 79 |
| 21.379 | 1,2-Benzenediol, 3-methoxy- | - | - | - | - | - | - | 0.20 | 76 | - | - |
| 21.432 | 1,2-Benzenediol, 4-methyl- | 0.74 | 63 | 1.05 | 77 | 0.72 | 80 | 0.79 | 65 | 0.79 | 79 |
| 22.243 | 2-Methoxy-4-vinylphenol | 0.36 | 76 | 1.02 | 85 | - |  | 0.96 | 87 |  |  |
| 22.246 | 2-Methoxy-4-vinylphenol | 0.40 | 85 | - | - | - | - | 0.97 | 88 | 0.27 | 85 |
| 22.248 | Ethanone, 1-(2-hydroxy-5-methylphenyl)- | 0.07 |  | - | - | 0.51 | 82 | - | - | 0.03 |  |
| 24.008 | 4-Ethylcatechol | - | - | 0.26 | 79 | 0.38 | 84 | 0.10 | 81 | 0.02 | 80 |
| 24.009 | 1,3-Benzenediol, 4-ethyl- | 0.04 | 65 | - | - | - | - | - | - | - | - |
| 24.127 | 1,3-Benzenediol, 2-methyl- | 0.13 | 79 | - | - | - | - | - | - | - | - |
| 24.134 | Orcinol | 0.18 | 82 | 0.24 | 78 | - |  | 0.19 | 80 | 0.17 |  |
| 24.687 | 1,2,3-Benzenetriol | 1.76 | 80 | - | - | - | - | 1.96 | 91 | 3.38 | 90 |
| 27.187 | 1,3-Benzenediol, 5-pentyl- | 0.06 | 87 | - | - | - | - | - | - | 0.12 | 70 |
| 38.157 | Acetamide, N-(4-ethoxy-3-hydroxyphenyl)- | - | - | - |  | - |  | - |  | 0.13 | 70 |
| 45.624 | Phenol, 2,2'-methylenebis[6-(1,1-dimethylethyl)-4-methyl- | - | - | 3.19 | 91 | - | - | 4.42 | 89 | - | - |
|  | **SUM** | **6.79** |  | **9.95** |  | **4.22** |  | **14.1** |  | **6.74** |  |
| **PHTHALIC ACID AND THEIR DERIVATIVES** | | | | | | | | | | | |
| 37.093 | Dibutyl phthalate | 0.37 | 85 | - | - | - | - | - | - | - | - |
| 37.096 | Phthalic acid, butyl hept-4-yl ester | - | - | - | - | - | - | 0.16 | 87 | - | - |
| 45.807 | Bis(2-ethylhexyl) phthalate | - | - | 0.25 | 72 | - | - | - | - | - | - |
|  | **SUM** | **0.37** |  | **0.25** |  | **0** |  | **0.16** |  | **0** |  |
| **NITROGEN-CONTAINING COMPOUNDS** | | | | | | | | | | | |
| 10.441 | Pyridine, 1,2,5,6-tetrahydro-1,2-dimethyl- | - | - | 0.69 | 67 | - | - | - | - | - | - |
| 11.444 | Urea, 1-methylcyclopropyl- | 0.13 | 73 | 0.94 | 73 | - | - | 1.03 | 75 | - |  |
| 14.374 | 2,4-Imidazolidinedione, 1-methyl- | - | - | - | - | - | - | - | - | 0.14 | 75 |
| 14.427 | 1-Methyl-4-amino-4,5(1H)-dihydro-1,2,4-triazole-5-one | - | - | 0,46 | 66 | - |  | - | - | - | - |
| 31.015 | 3-Methyl-4-phenyl-1H-pyrrole | - | - | 0.46 | 86 | - | - | - | - | - | - |
|  | **SUM** | **0.13** |  | **2.55** |  | **0** |  | **1.03** |  | **0.14** |  |
| **UBIQUINONES** | | | | | | | | | | | |
| 44.657 | γ-tocopherol | 0.38 | 65 | 3.04 | 84 | - |  | 0.57 | 85 | - |  |
|  | **SUM** | **0.38** |  | **3.04** |  | **0** |  | **0.57** |  | **0** |  |
| **TERPENES** | | | | | | | | | | | |
| 28.848 | 2-Hexadecene, 3,7,11,15-tetramethyl-, [R-[R*,R*-(E)]]- | - | - | - | - | - | - | - |  | 0.03 | 74 |
| 29.113 | Phytol, acetate | - | - | - | - | - | - | - | - | 0.11 | 80 |
| 29.117 | 3,7,11,15-Tetramethyl-2-hexadecen-1-ol | 0.92 | 84 | 0.71 | 87 | 0.32 | 80 | 0.15 | 79 | 0.54 | 85 |
| 36.101 | Phytol | 12.44 | 94 | 14.24 | 93 | 12.58 | 94 | 13.26 | 93 | 10.96 | 94 |
| 46.133 | Friedelan-3-one | - | - | - | - | - | - | - | - | 0.20 | 63 |
| 46.157 | 6a,14a-Methanopicene, perhydro-1,2,4a,6b,9,9,12a-heptamethyl-10-hydroxy- | 0.79 | 69 | - | - | 4.36 | 70 | 1.60 | 72 | - | - |
| 47.853 | Squalene | 0.27 | 73 | 0.22 | 65 | - | - | - | - | - | - |
| 51.341 | α-Amyrin | 0.77 | 68 | - | - | 1.73 | 70 | 0.70 | 65 | - | - |
| 51.714 | Lupeol | 2.47 | 66 | - | - | 2.47 | 79 | 1.86 | 76 | - | - |
|  | **SUM** | **17.7** |  | **15.2** |  | **21.5** |  | **17.6** |  | **11.8** |  |
| **STEROLS** | | | | | | | | | | | |
| 46.258 | Cholesterol | - | - | - | - | - | - | 0.13 | 68 | - | - |
| 50.368 | Campesterol | 0.55 | 75 | 1,26 | 61 | - | - | 0.28 | 71 | 0.36 | 74 |
| 51.332 | Stigmasterol | 4.02 | 71 | 2,19 | 79 | - | - | 0.67 | 75 | 2.08 | 74 |
|  | **SUM** | **4.57** |  | **3.45** |  | **0** |  | **1.08** |  | **2.44** |  |

Notes: IP – identification probability; RT – retention time.

**Supplementary Table 2**. Variation in secondary metabolite content in *R. linearifolia* flowers at different altitudes.

| **RT, min** | **Metabolites** | **MED** | | **YT** | | **KET** | | **QUR** | | **BAU** | |  |
| --- | --- | --- | --- | --- | --- | --- | --- | --- | --- | --- | --- | --- |
|  |  | Content, % | IP, % | Content, % | IP, % | Content, % | IP, % | Content, % | IP, % | Content, % | IP, % |  |
| **FATTY ACIDS** | | | | | | | | | | | | |
| 33.697 | Hexadecanoic acid | 1.16 | 87 | - | - | - | - | - | - | 4.82 | 79 |  |
| 37.866 | 9,12-Octadecadienoic acid (Z,Z)- | 2.28 | 86 | - | - | - | - | - | - | 2.88 | 92 |  |
| 38.422 | 9,12,15-Octadecatrienoic acid, (Z,Z,Z)- | 2.66 | 91 | - | - | 0.69 | 63 | 0.42 | 83 | 3.06 | 90 |  |
|  | **SUM** | **6.1** |  | **0** | **-** | **0.69** |  | **0.42** |  | **10.76** |  |  |
| **FATTY ACID ESTERS** | | | | | | | | | | | | |
| 24.651 | 2-Octenoic acid, 4,5,7-trhydroxy | - | - | - | - | 1.69 | 67 | - | - | - | - |  |
| 29.555 | Tetradecanoic acid | 0.03 | 65 | - | - | - | - | - | - | 0.01 | 65 |  |
| 29.724 | Tetradecanoic acid, ethyl ester | 0.26 | 65 | - | - | - | - | - | - | 0.04 | 64 |  |
| 30.008 | Isopropyl myristate | 0.06 | 67 | - | - | - | - | - | - | - | - |  |
| 33.712 | Hexadecanoic acid, ethyl ester | 6.13 | 91 | - | - | 4.49 | 90 | 3.70 | 89 | 1.66 | 89 |  |
| 37.357 | Octadecanoic acid, ethyl ester | 0.43 | 83 | - | - | - | - | - | - | 0.08 | 66 |  |
| 37.398 | Ethyl Oleate | 0.78 | 80 | - | - | - | - | - | - | 0.43 | 71 |  |
| 37.399 | (E)-9-Octadecenoic acid ethyl ester | 0.09 | 75 | - | - | - | - | - | - | - | - |  |
| 37.746 | 9,12-Octadecadienoic acid, ethyl ester  (linoleic acid ester) | 5.99 | 92 | 4.61 | 92 | 2.95 | 88 | 2.55 | 92 | 2.50 | 91 |  |
| 38.276 | 9,12,15-Octadecatrienoic acid, ethyl ester, (*Z,Z,Z*)- | 8.47 | 95 | - | - | - | - | - | - | 3.97 | 91 |  |
| 38.278 | Ethyl 9,12,15-octadecatrienoate | - | - | 6.49 | 92 | 6.3 | 91 | 2.56 | 89 | - | - |  |
| 40.707 | Methyl 19-methyl-eicosanoate | 0.04 | 75 | - | - | - | - | - | - | - | - |  |
| 40.761 | Hexadecanoic acid, 1-(hydroxymethyl)-1,2-ethanediyl ester | 0.04 | 67 | - | - | - | - | - | - | 0.21 | 69 |  |
| 41.828 | Hexanedioic acid, bis(2-ethylhexyl) ester | - | - | - | - | - | - | 0.39 | 73 | - | - |  |
| 45.168 | Hexadecanoic acid, 2-hydroxy-1-(hydroxymethyl)ethyl ester | 5.65 | 88 | - | - | - | - | - | - | 4.90 | 87 |  |
| 42.562 | Myristic acid, phenyl ester | - | - | - | - | - | - | 5.47 | 68 | - | - |  |
| 45.622 | Phenyl palmitate | - | - | 2.72 | 67 | - | - | 6.05 | 73 | 0.30 | 71 |  |
| 45.927 | Tetracosanoic acid, methyl ester | - | - | - | - | - | - | - | - | 0.08 | 69 |  |
| 46.475 | Eicosanoic acid, octadecyl ester | - | - | - | - | - | - | - | - | 0.48 | 67 |  |
| 48.151 | Octadecanoic acid, 2-hydroxy-1-(hydroxymethyl)ethyl ester | - | - | - | - | - | - | - | - | 0.09 | 63 |  |
| 48.658 | 9,12-Octadecadienoic acid (Z,Z)-, 2-hydroxy-1-(hydroxymethyl)ethyl ester | 1.11 | 72 | - | - | - | - | - | - | 3.65 | 86 |  |
| 48.644 | 9,12-Octadecadienoic acid (Z,Z)-, 2,3-dihydroxypropyl ester | 1.03 | 82 |  |  |  |  |  |  | 0.44 | 81 |  |
| 49.207 | Butyl 9,12,15-octadecatrienoate | 0.61 | 84 | - | - | - | - | - | - | - | - |  |
| 49.208 | 9,12,15-Octadecatrienoic acid, 2-phenyl-1,3-dioxan-5-yl ester | - | - | - | - | - | - | - | - | 0.10 | 63 |  |
| 49.242 | Linolenic acid, 2-hydroxy-1-(hydroxymethyl)ethyl ester (Z,Z,Z)- | 3.33 | 74 | - | - | - | - | - | - | 3.87 | 75 |  |
| 49.249 | 9,12,15-Octadecatrienoic acid, 2,3-dihydroxypropyl ester, (Z,Z,Z)- | - | - | - | - | - | - | - | - | 0.73 | 72 |  |
|  | **SUM** | **34.1** |  | **13.8** |  | **15.4** |  | **20.7** |  | **23.5** | **-** |  |
| **CARBOHYDRATES AND THEIR DERIVATIVES** | | | | | | | | | | | | |
| 21.211 | 1,4:3,6-Dianhydro-α-d-glucopyranose |  |  |  |  | 1.07 | 78 | - | - | 0.05 | 72 |  |
| 26.497 | α-d-Lyxofuranoside, methyl | 0.12 | 71 | - | - | - | - | - | - | 0.23 | 70 |  |
| 28.034 | Sucrose | 0.27 | 73 | - | - | 2.72 | 73 | 1.43 | 74 | 0.53 | 72 |  |
| 29.618 | *β*-*D*-Glucopyranose, 1,6-anhydro- | 0.24 | 85 | - | - | - | - | 1.81 | 86 | 0.71 | 85 |  |
| 29.622 | D-Allose | 0.54 | 85 | 3.22 | 88 | 6.5 | 88 | - | - | 1.10 | 88 |  |
| 31.512 | Ethyl *α*-*D*-glucopyranoside | 14.73 | 86 | 13.99 | 84 | 16.72 | 84 | 9.44 | 84 | 13.85 | 84 |  |
| 33.591 | β-D-Glucopyranose, 4-O-β-D-galactopyranosyl- | - | - | - | - | - | - | 0.92 | 75 | 0.08 | 65 |  |
| 33.599 | α-D-Galactopyranoside, methyl | 0.34 | 76 | - | - | - | - | - | - | 0.13 | 74 |  |
| 42.694 | α-Methyl-D-mannopyranoside | 0.60 | 65 | - | - | - | - | - | - | 1.84 | 70 |  |
| 42.716 | α-D-Glucopyranose, 4-O-β-D-galactopyranosyl- | - | - | 0.6 | 68 | - | - | 2.25 | 65 | 1.32 | 69 |  |
| 45.057 | α-d-Glucofuranosyl benzenesulfonate | - | - | - | - | - | - | 7.67 | 65 | - |  |  |
|  | **SUM** | **16.8** |  | **17.8** |  | **27.0** |  | **23.5** |  | **19.8** |  |  |
| **ALKANES** | | | | | | | | | | | | |
| 12.708 | Dodecane | 0.87 | 76 | - | - | - | - | - | - | - | - |  |
| 18.079 | Tetradecane | 0.40 | 93 | - | - | 0.51 | 82 | - | - | - | - |  |
| 20.650 | Pentadecane | - |  | - | - | 1.07 | 77 | - | - | - | - |  |
| 23.097 | Hexadecane | 0.03 | 80 | - | - | 0.66 | 73 | - | - | - | - |  |
| 36.379 | Eicosane | - |  | - | - | - | - | - | - | 0.17 | 90 |  |
| 37.329 | Heneicosane | - |  | 4.35 | 89 | - | - | - | - | 0.81 | 91 |  |
| 40.649 | Pentacosane | - |  | - | - | - | - | - | - | 0.09 | 75 |  |
| 43.719 | Tetratetracontane | - |  | - | - | - | - | - | - | 0.21 | 65 |  |
|  | **SUM** | **1.30** |  | **4.35** |  | **4.35** |  | **0** |  | **1.28** |  |  |
| **ALKЕNES** | | | | | | | | | | | | |
| 18.391 | 1-Tetradecene | 0.04 | 75 | - | - | - | - | - | - | - | - |  |
|  | **SUM** | **0.44** |  | **0** |  | **0** |  | **0** |  | **0** |  |  |
| **NITRILES** | | | | | | | | | | | | |
| 6.907 | 3-Butenenitrile, 2-methyl- | - | - | - | - | - | - | - | - | 1.17 | 79 |  |
| 6.917 | Butanenitrile, 2-methylene- | - | - | - | - | - | - | - | - | 0.25 | 65 |  |
| 10.729 | Propanenitrile, 3-methoxy- | 0.08 | 65 | - | - | - | - | - | - | 0.10 | 67 |  |
| 27.945 | 1-Isoquinolinecarbonitrile | - | - | - | - | 0.47 | 67 | - | - | 0.04 | 75 |  |
|  | **SUM** | **0.08** |  | **0** |  | **0.47** |  | **0** |  | **1.56** |  |  |
| **ALCOHOLS, ALDEHYDES, KETONES, ESTERS AND THEIR DERIVATIVES** | | | | | | | | | | | | |
| 6.721 | Glycolaldehyde dimethyl acetal | 0.86 | 67 | - | - | - | - | - | - | - | - |  |
| 7.116 | 1,2-Ethanediol, monoacetate | 0.15 | 80 | - | - | - | - | - | - | - | - |  |
| 9.323 | 1,2-Ethanediol, diacetate | 0.08 | 82 | - | - | - | - | - | - | 0.06 | 80 |  |
| 9.325 | 2-Propanone, 1-(acetyloxy)- | 0.07 | 78 | - | - | - | - | - | - | 0.06 | 76 |  |
| 12.516 | Glycerin | 1.88 | 76 | - | - | - | - | - | - | 2.64 | 81 |  |
| 14.678 | Benzeneacetaldehyde | 0.15 | 88 | - | - | 0.91 | 81 | 1.44 | 93 | 0.08 | 90 |  |
| 15.071 | 5-Hexen-2-one | 0.30 | 77 | - | - | - | - | - | - | - | - |  |
| 18.194 | Cyclopropyl carbinol | 0.73 | 72 | - | - | - | - | - | - | 0.50 | 75 |  |
| 18.205 | Pentanal | 0.33 | 75 | - | - | - | - | 0.59 | 69 | 0.53 | 71 |  |
| 21.048 | 1-Butanol, 3-methyl-, acetate | 0.13 | 66 | - | - | - | - | - | - | - | - |  |
| 21.063 | 1-Butanol, 3-methyl-, acetate | - | - | - | - | - | - | - | - | 0.05 | 66 |  |
| 21.695 | Allyl acetate | 0.06 | 67 | - | - | - | - | - | - | - | - |  |
| 21.701 | 5,9-Dodecadien-2-one, 6,10-dimethyl-, (E,E))- | - | - | - | - | - | - | - | - | 0.03 | 68 |  |
| 22.244 | Ethanone, 1-(2-hydroxy-5-methylphenyl)- | 0.06 | 73 | - | - | - | - | - | - | 0.03 | 82 |  |
| 22.559 | 2,7-Octadiene-1,6-diol, 2,6-dimethyl- | 0.02 | 69 | - | - | - | - | - | - | 0.04 | 77 |  |
| 28.326 | Pentaethylene glycol | - | - | - | - | - | - | - | - | 0.03 | 68 |  |
| 28.756 | Ethanol, 2-(dodecyloxy)- | - | - | - | - | - | - | 0.61 | 78 | - | - |  |
| 29.115 | 3,7,11,15-Tetramethyl-2-hexadecen-1-ol | 0.06 | 71 | 0,94 | 79 | - | - | - | - | 0,23 | 83 |  |
| 34.560 | Octadecanal | - | - | 0.91 | 78 |  |  | 0.54 | 74 | - | - |  |
| 34.561 | 17-Octadecenal | - | - | - | - | - | - | - | - | 0.03 | 68 |  |
| 34.562 | Ethanol, 2-(9-octadecenyloxy)-, (Z)- | - | - | - | - | - | - | - | - | 0.07 | 67 |  |
| 34.982 | Ethanone, 1-(2,6-dihydroxy-4-methoxyphenyl)- | 0.12 | 71 | - | - | - | - | - | - | - | - |  |
|  | **SUM** | **5.00** |  | **1.85** |  | **0.91** |  | **3.18** |  | **4.38** |  |  |
| **CYCLIC KETONES, LACTONES AND THEIR DERIVATIVES** | | | | | | | | | | | | |
| 10.457 | 4-Cyclopentene-1,3-dione | 0.48 | 70 | - | - | - | - | - | - | 0.42 | 77 |  |
| 11.027 | 2-Cyclopenten-1-one, 2-hydroxy- | 0.57 | 90 | - | - | - | - | - | - | 0.19 | 88 |  |
| 13.842 | 2-Cyclopenten-1-one, 2-hydroxy-3-methyl- | 0.80 | 71 | - | - | - | - | - | - | 0.72 | 80 |  |
| 11.035 | 1,2-Cyclopentanedione | 1.13 | 92 | - | - | 2.44 | 83 | 1.23 | 73 | 1.66 | 87 |  |
| 13.846 | 1,2-Cyclopentanedione, 3-methyl- | - | - | - | - | - | - | - | - | 0.26 | 81 |  |
| 15.063 | Cyclopentane, 1-acetyl-1,2-epoxy- | - | - | - | - | - | - | - | - | 0.48 | 67 |  |
| 19.184 | 2,4-Cycloheptadien-1-one, 2,6,6-trimethyl- | - |  | 0.79 | 81 | - | - | - | - | - | - |  |
| 21.618 | (*S*)-(+)-2',3'-Dideoxyribonolactone | 0.57 | 84 | - | - | - | - | - | - | 0.69 | 77 |  |
| 27.187 | 1,8(2H,5H)-Naphthalenedione, hexahydro-8a-methyl-, cis- | - | - | - | - | - | - | - | - | 0.03 | 67 |  |
| 32.221 | α,β-Gluco-octonic acid lactone | - | - | - | - | - | - | - | - | 1.30 | 66 |  |
| 32.616 | 3-Deoxy-d-mannoic lactone | - | - | - | - | - | - | - | - | 0.05 | 67 |  |
| 44.444 | 1-Cyclohexanone, 2-methyl-2-(3-methyl-2-oxobutyl) | 2.35 | 64 | 32.9 | 69 | 14.87 | 65 | 22.27 | 68 | 1.64 | 66 |  |
|  | **SUM** | **5.90** |  | **33.7** |  | **17.3** |  | **23.5** |  | **7.44** |  |  |
| **CARBOXYLIC ACID AND THEIR DERIVATIVES** | | | | | | | | | | | | |
| 6.921 | Propanoic acid, 3-(acetylthio)-2-methyl- | - | - | - | - | - | - | - | - | 0.26 | 67 |  |
| 6.947 | Acetic acid, (acetyloxy)- | 0.75 | 70 | - | - | - | - | - | - | - | - |  |
| 7.553 | Propanoic acid, 2-oxo-, methyl ester | 3.80 | 83 | - | - | 2.54 | 69 | 2.11 | 75 | 3.16 | 79 |  |
| 11.739 | Carbamic acid, methyl-, phenyl ester | - | - | - | - | - | - | - | - | 0.05 | 75 |  |
| 15.438 | Propanoic acid, 3-(acetylthio)-2-methyl-, (S)- | - | - | - | - | - | - | - | - | 0.05 | 67 |  |
| 15.459 | Methyl acetoxyacetate | 0.30 |  | - | - | - | - | - | - | 0.23 | 70 |  |
| 21.086 | Acetic acid, pentyl ester | 0.04 | 67 | - | - | - | - | - | - | - | - |  |
| 24.653 | Ethyl trans-3-methyl-2-oxiranecarboxylate | - | - | - | - | - | - | 1.80 | 72 | - | - |  |
| 25.316 | Ethyl 2,3-epoxybutyrate | 0.08 | 68 | - | - | - | - | 0.57 | 66 | 0.24 | 69 |  |
| 27.021 | 2-Propenoic acid, 2-methyl-, hexyl ester | - | - | - | - | - | - | - | - | 0.06 | 66 |  |
|  | **SUM** | **4.97** |  | **0** |  | **2.54** |  | **4.48** |  | **3.79** |  |  |
| **PHOSPHORIC ACID ESTERS** | | | | | | | | | | | | |
| 26.314 | Phosphoric acid, diethyl nonyl ester | 0.03 | 70 | - | - | - | - | - | - | 0.06 | 66 |  |
| 26.318 | Phosphoric acid, diethyl dodecyl ester | - | - | - | - | - | - | - | - | 0.07 | 73 |  |
| 26.319 | Phosphoric acid, diethyl octyl ester | - | - | - | - | - | - | - | - | 0.11 | 73 |  |
|  | **SUM** | **0.03** |  | **0** |  | **0** |  | **0** |  | **0.24** |  |  |
| **FURAN AND PYRAN DERIVATIVES** | | | | | | | | | | | | |
| 8.554 | 2-Furanmethanol | 0.06 | 87 | - | - | - | - | - | - | - | - |  |
| 12.698 | 2(5H)-Furanone | 0.77 | 68 | - | - | - | - | - | - | 0.78 | 66 |  |
| 13.099 | Ethyl 2-(5-methyl-5-vinyltetrahydrofuran-2-yl)propan-2-yl carbonate | 0.65 | 91 | - | - | 6.5 | 88 | 3.52 | 84 | 1.63 | 90 |  |
| 14.895 | 2-Hydroxy-gamma-butyrolactone | 3.11 | 89 | - | - | 1.44 | 82 | 1.15 | 78 | 3.57 | 86 |  |
| 15.045 | Maltol | 0.09 | 68 | - | - | - | - | 1.16 | 71 | 0.06 | 70 |  |
| 16.111 | 2,5-Dimethyl-4-hydroxy-3(2H)-furanone | 0.14 | 76 | - | - | 2.53 | 77 | 1.13 | 76 | 0.38 | 70 |  |
| 16.353 | 2H-Pyran-3-ol, 6-ethenyltetrahydro-2,2,6-trimethyl- | 0.66 | 70 | 1.92 | 86 | 3.72 | 86 | 0.87 | 78 | 1.08 | 87 |  |
| 17.423 | 4H-Pyran-4-one, 2,3-dihydro-3,5-dihydroxy-6-methyl- | 0.26 | 68 | - | - | - | - | - | - | 0.25 | 65 |  |
| 19.335 | Benzofuran, 2,3-dihydro- | 0.35 | 85 | 1.71 | 82 | 2.05 | 80 | 0.7 | 83 | 0.48 | 80 |  |
| 19.535 | 2(3H)-Furanone, 5-acetyldihydro- | 0.15 | 83 | - | - | - | - | - | - | 0.21 | 84 |  |
| 22.109 | l-Alanine, N-(2-furoyl)-, ethyl ester | 0.02 | 74 | . | . | - | - | - | - | - | - |  |
| 23.486 | trans-Linalool oxide (furanoid) | 0.51 | 76 | 6.89 | 82 | 2.94 | 78 | - | - | 0.54 | 80 |  |
| 23.494 | 2-Furanmethanol, 5-ethenyltetrahydro-α,α,5-trimethyl-, cis- | 0.31 | 77 | - | - | - | - | - | - | 0.40 | 76 |  |
| 26.644 | 1,6-Anhydro-2,3-dideoxy-β-D-threo-hexopyranose | - | - | - | - | - | - | - | - | 0.12 | 65 |  |
| 27.052 | 1,6-Anhydro-3,4-dideoxy-β-D-gluco-hexopyranose | 0.04 | 65 | - | - | - | - | - | - | 0.09 | 68 |  |
|  | **SUM** | **7.12** |  | **10.52** |  | **19.18** |  | **8.53** |  | **9.59** |  |  |
| **PHENOLIC COMPOUNDS** | | | | | | | | | | | | |
| 11.779 | Phenol | 0.13 | 81 | 1.78 | 86 | 3.36 | 81 | 0.87 | 92 | 0.67 | 72 |  |
| 15.643 | Phenol, 2-methoxy- | 0.05 | 66 | - | - | - | - | - | - | 0.05 | 79 |  |
| 18.768 | Catechol | 1.63 | 91 | - | - | 1.99 | 88 | 1.06 | 90 | 1.55 | 89 |  |
| 20.371 | Phenol, 2,4,6-trimethyl- | - | - | - | - | - | - | 0.29 | 87 | - | - |  |
| 20.688 | 1,2-Benzenediol, 3-methyl- | 0.33 | 82 | - | - | - | - | - | - | 0.50 | 84 |  |
| 21.369 | 1,4-Benzenediol, 2-methoxy- | 0.11 | 79 | - | - | - | - | - | - | - | - |  |
| 21.377 | 1,2-Benzenediol, 3-methoxy- | 0.10 | 90 | - | - | 1.18 | 75 | - | - | - | - |  |
| 21.427 | 1,2-Benzenediol, 4-methyl- | 0.50 | 80 | - | - | 0.97 | 71 | - | - | 0.76 | 78 |  |
| 22.242 | 2-Methoxy-4-vinylphenol | 0.13 | 84 | - | - | 1.15 | 85 | 0.73 | 89 | 0.23 | 80 |  |
| 22.244 | Ethanone, 1-(2-hydroxy-5-methylphenyl)- | - | - | 1.35 | 88 | - | - | - | - | - | - |  |
| 24.005 | 4-Ethylcatechol | - | - | - | - | - | - | - | - | 0.08 | 68 |  |
| 24.009 | 1,3-Benzenediol, 4-ethyl- | - | - | - | - | - | - | - | - | 0.03 | 72 |  |
| 24.127 | Orcinol | 0.04 | 82 | - | - | - | - | - | - | 0.14 | 78 |  |
| 24.418 | Phenol, 2,6-dimethoxy- | 0.05 | 88 | - | - | - | - | 0.47 | 92 | - | - |  |
| 24.683 | 1,2,3-Benzenetriol | 4.76 | 92 | - | - | - | - | - | - | 5.04 | 88 |  |
| 25.289 | Phenol, 2,4-bis(1,1-dimethylethyl)- | - | - | - | - | 1.34 | 74 | - | - | - | - |  |
| 27.188 | 1,3-Benzenediol, 5-pentyl- | - | - | - | - | - | - | - | - | 0.09 | 65 |  |
| 27.391 | Benzoic acid, 3-ethyloxy-, ethyl ester | - | - | - | - | - | - | 0.43 | 68 | - | - |  |
|  | **SUM** | **7.83** |  | **3.13** |  | **9.99** |  | **3.85** |  | **9.14** |  |  |
| **PHTHALIC ACID AND THEIR DERIVATIVES** | | | | | | | | | | | | |
| 37.089 | Dibutyl phthalate | 0.09 | 78 | - | - | - | - | - | - | - | - |  |
| 45.804 | Bis(2-ethylhexyl) phthalate | - | - | - | - | 3.01 | 79 | - | - | - | - |  |
| 45.806 | Diisooctyl phthalate | - | - | 4.19 | 74 | - | - | 1.61 | 83 | - | - |  |
|  | **SUM** | **0.09** |  | **4.19** |  | **3.01** |  | **1.61** |  | **0** |  |  |
| **DIOXOLANE DERIVATIVE** | | | | | | | | | | | | |
| 14.422 | 1,3-Dioxol-2-one,4,5-dimethyl- | 1.15 | 68 | - | - | - | - | - | - | 0.25 | 69 |  |
| 25.183 | 2-t-Butyl-4-methyl-5-oxo-[1,3]dioxolane-4-carboxylic acid | - | - | - | - | - | - | - | - | 0.16 | 66 |  |
| 25.262 | 1,3-Dioxolane, 2-ethyl- | 0.28 | 67 | - | - | - | - | - | - | 0.08 | 68 |  |
|  | **SUM** | **1.42** |  | **0** |  | **0** |  | **0** |  | **0.49** |  |  |
| **1,4-DIOXIN DERIVATIVES** | | | | | | | | | | | | |
| 15.075 | 1,4-Dioxin, 2,3-dihydro-5,6-dimethyl- | 0.37 | 75 | - | - | - | - | - | - | 0.14 | 71 |  |
|  | **SUM** | **0.37** |  | **0** |  | **0** |  | **0** |  | **0.14** |  |  |
| **DIOXEPINE DERIVATIVES** | | | | | | | | | | | | |
| 14.359 | 5H-1,4-Dioxepin, 2,3-dihydro-7-methyl- | - | - | - | - | - | - | 3.36 | 69 | 0.74 |  |  |
|  | **SUM** | **0** |  | **0** |  | **0** |  | **3.36** |  | **0.74** |  |  |
| **NITROGEN-CONTAINING COMPOUNDS** | | | | | | | | | | | | |
| 14.370 | 2,5-Piperazinedione | - | - | - | - | - | - | - | - | 0.27 | 76 |  |
| 14,377 | 1-Methyl-4-amino-4,5(1H)-dihydro-1,2,4-triazole-5-one | 0.32 | 69 | - | - | - | - | - | - | - | - |  |
| 16.112 | 2,4(1H,3H)-Pyrimidinedione, 5-hydroxy- | - | - | 1.36 | 74 | - | - | - | - | - | - |  |
| 16.114 | 2,4,5-Trihydroxypyrimidine | - | - | - | - | - | - | - | - | 0.33 | 75 |  |
| 27.550 | 2-Pyrrolidinecarboxylic acid-5-oxo-, ethyl ester | 0.05 | 67 | - | - | 0.52 | 65 | 0.76 | 87 | - | - |  |
| 27.939 | 6-Cyanoquinoline | 0.03 | 80 | - | - | - | - | - | - | - | - |  |
| 38.158 | Acetamide, N-(4-ethoxy-3-hydroxyphenyl)- | - | - | - | - | - |  | - | - | 0.14 | 70 |  |
|  | **SUM** | **0.4** |  | **1.36** |  | **0.52** |  | **0.76** |  | **0.74** |  |  |
| **TERPENES** | | | | | | | | | | | | |
| 23.561 | α-Methyl-α-[4-methyl-3-pentenyl]oxiranemethanol | 0.21 | 78 | - | - | - | - | 2.55 | 71 | 0.30 | 81 |  |
| 29.112 | Phytol, acetate | 0.04 | 78 | - | - | 0.70 | 72 | - | - | 0.06 | 73 |  |
| 34.460 | Cembrene | - | - | - | - | - | - | - | - | 0.05 | 74 |  |
| 34.461 | 1,3,6,10-Cyclotetradecatetraene, 14-isopropyl-3,7,11-trimethyl-, (+)- | - | - | - | - | - | - | - | - | 0.13 | 81 |  |
| 36.089 | Phytol | 2.46 | 93 | 1.25 | 89 | - | - | 0.62 | 86 | 2.66 | 93 |  |
| 46.581 | 6-O-Acetyl-1-[[4-bromophenyl]thio]-β-d-glucoside S,S-dioxide | - | - | 5.82 | 63 | - | - | 2.90 | 63 | - | - |  |
| 47.453 | β-Amyrin | 0.19 | 65 | - | - | - | - | - | - | - | - |  |
| 47.842 | Supraene | 0.06 | 63 | - | - | - | - | - | - | - | - |  |
| 47.845 | Squalene | 0.37 | 74 | - | - | - | - | - | - | - | - |  |
| 51.291 | 13,27-Cycloursan-3-ol, acetate, (3β,13β,14β)- | - | - | - | - | - | - | - | - | 0.39 | 72 |  |
| 51.301 | α-Amyrin | 1.48 | 80 | - | - | - | - | - | - | - | - |  |
| 51.667 | Lupeol | 0.57 | 71 | - | - | - | - | - | - | - | - |  |
|  | **SUM** | **5.38** |  | **7.07** |  | **0.70** |  | **6.07** |  | **3.59** |  |  |
| **STEROLS** | | | | | | | | | | | | |
| 46.251 | Cholest-5-en-3-ol | 0.13 | 71 | - | - | - | - | - | - | - | - |  |
| 50.351 | Campesterol | 1.06 | 77 | - | - | - | - | - | - | 0.49 | 70 |  |
| 51.285 | Stigmasterol | 1.76 | 75 | - | - | - | - | - | - | 1.72 | 70 |  |
| 50.359 | Ergost-5-en-3-ol, (3β)- | - | - | - | - | - | - | - | - | 0.53 | 78 |  |
|  | **SUM** | **2.95** |  | **0** |  | **0** |  | **0** |  | **2.74** |  |  |
| **SILYL DERIVATIVES** | | | | | | | | | | | | |
| 22.169 | 1,1,3,3-Tetramethyl-1,3-disilaphenalane | 0.13 | 75 | - | - | - | - | - | - | - | - |  |
| 30.245 | 3,7,11,14,18-Pentaoxa-2,19-disilaeicosane, 2,2,19,19-tetramethyl- | - | - | 2.21 | 65 | - | - | - | - | - | - |  |
|  | **SUM** | **0.13** |  | **2.21** |  | **0** |  | **0** |  | **0** |  |  |

| **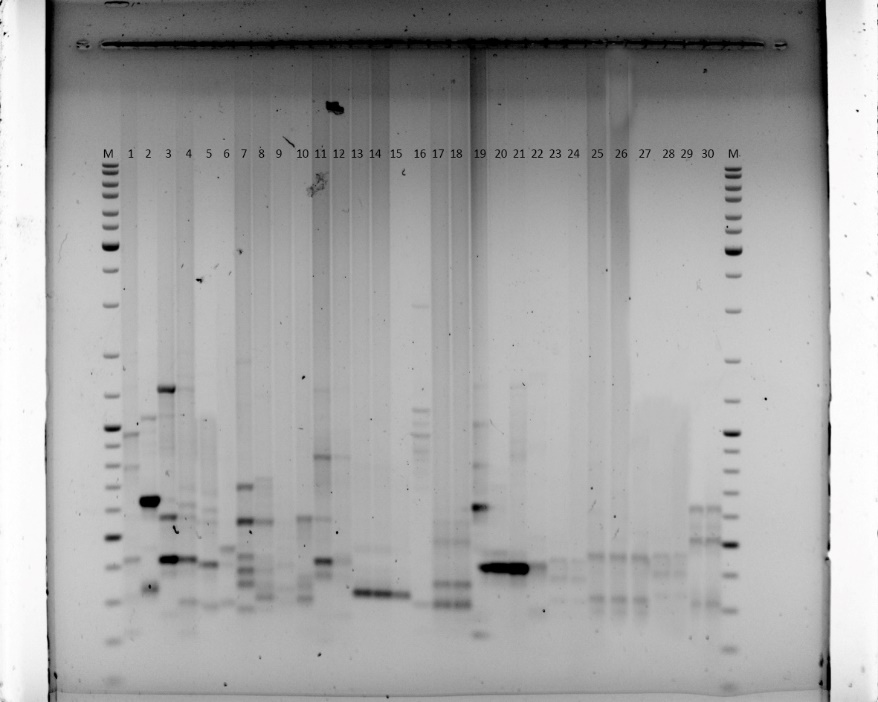** | **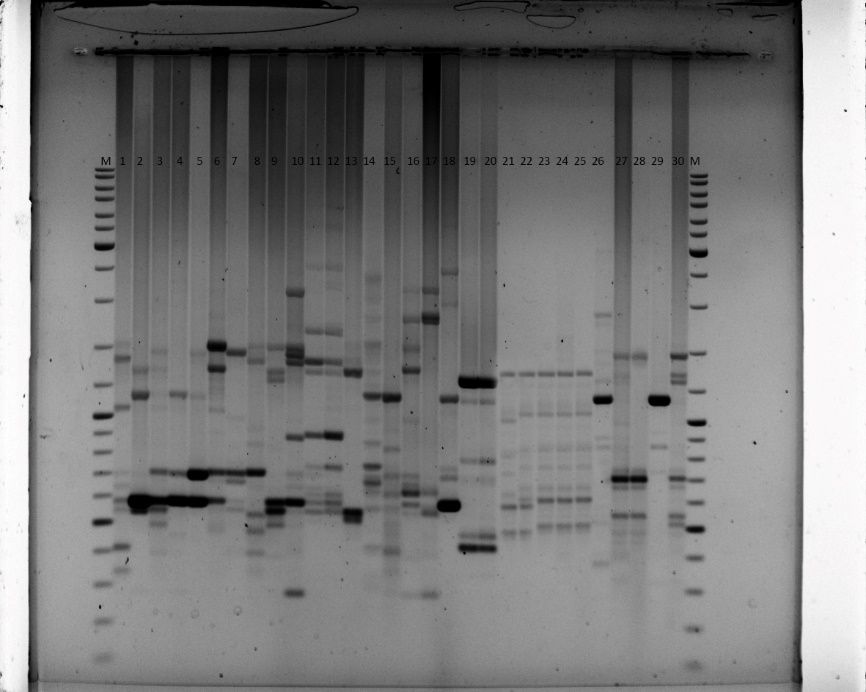** |
| --- | --- |
| **1813** | **1814** |

**Supplementary Figure 2.** The band profiles with ISSR primers 1813 (A) and 1814 (B) for the samples of *R. linearifolia*. The numbers on the top indicate the sample numbers (1‐30): 1-10 (BAU), 11- (QUR), 16-20 (MED), 21-25 (YT), 25-30 (KET). M—Thermo Scientific GeneRuler DNA Ladder Mix, (100–10,000 bp).


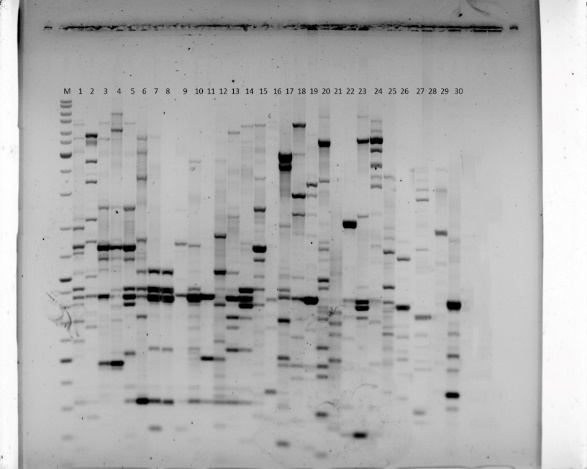


**Supplementary Figure 3.** The band profiles with iPBS primers 2221 for the samples of *R. linearifolia*. The numbers on the top indicate the sample numbers (1‐30): 1-10 (BAU), 11- (QUR), 16-20 (MED), 21-25 (YT), 25-30 (KET). M—Thermo Scientific GeneRuler DNA Ladder Mix, (100–10,000 bp).


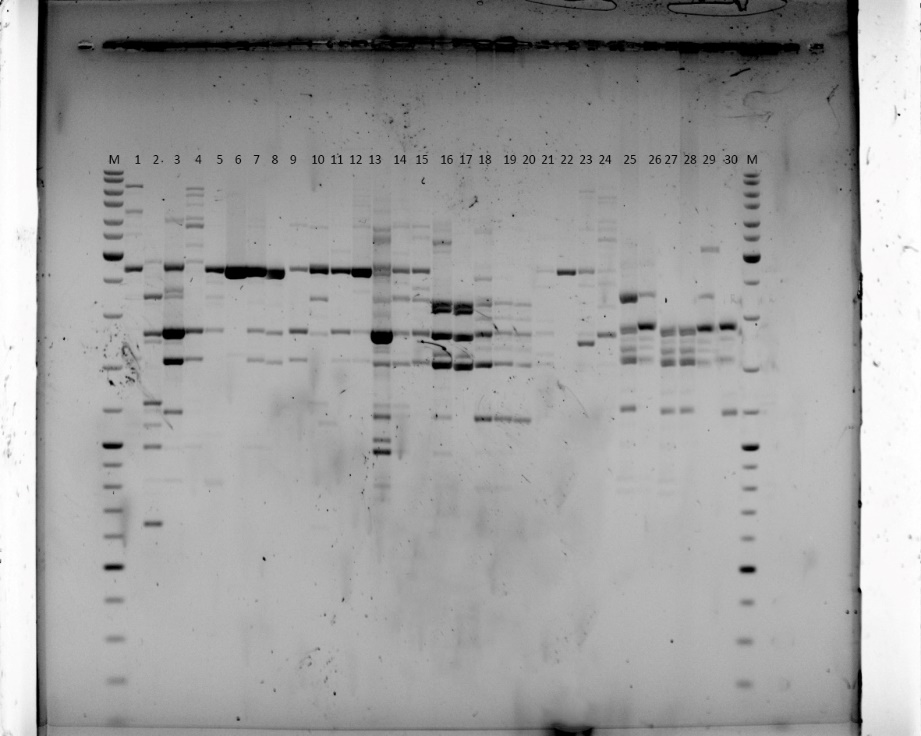


**Supplementary Figure 4.** The band profiles with iPBS primers 2228 for the samples of *R. linearifolia*. The numbers on the top indicate the sample numbers (1‐30): 1-10 (BAU), 11- (QUR), 16-20 (MED), 21-25 (YT), 25-30 (KET). M—Thermo Scientific GeneRuler DNA Ladder Mix, (100–10,000 bp).


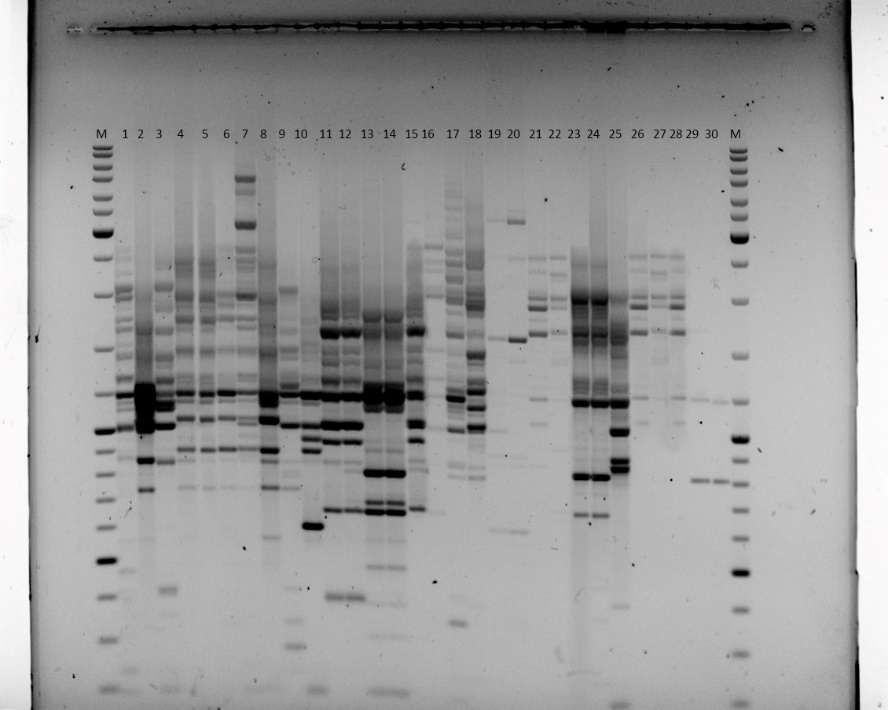


**Supplementary Figure 5.** The band profiles with iPBS primers 2230 for the samples of *R. linearifolia*. The numbers on the top indicate the sample numbers (1‐30): 1-10 (BAU), 11- (QUR), 16-20 (MED), 21-25 (YT), 25-30 (KET). M—Thermo Scientific GeneRuler DNA Ladder Mix, (100–10,000 bp).


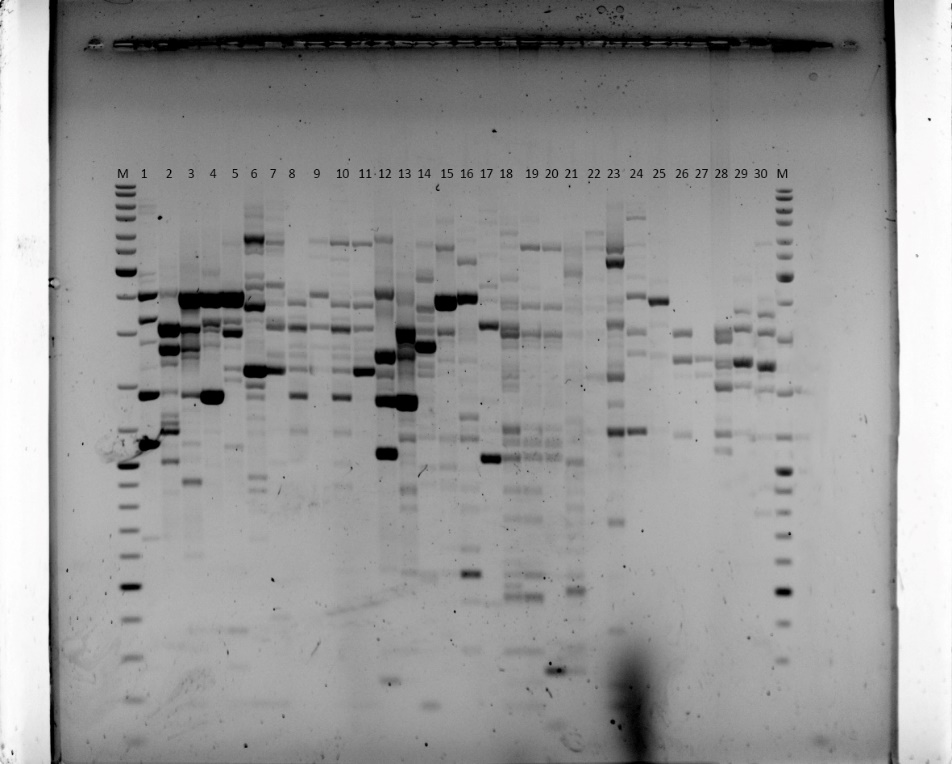


**Supplementary Figure 6.** The band profiles with iPBS primers 2232 for the samples of *R. linearifolia*. The numbers on the top indicate the sample numbers (1‐30): 1-10 (BAU), 11- (QUR), 16-20 (MED), 21-25 (YT), 25-30 (KET). M—Thermo Scientific GeneRuler DNA Ladder Mix, (100–10,000 bp).


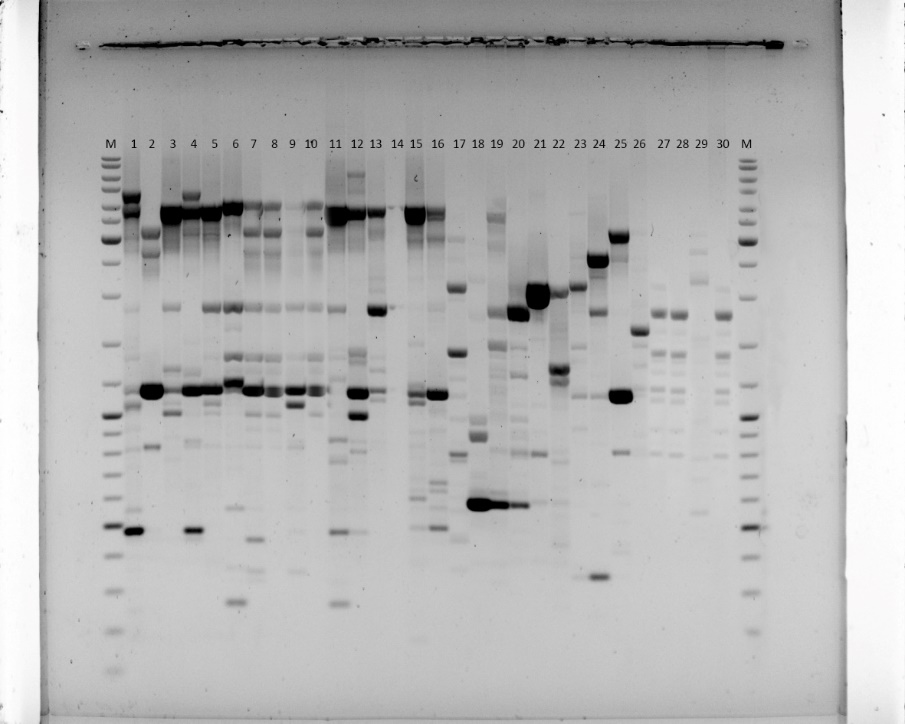


**Supplementary Figure 7.** The band profiles with iPBS primers 2240 for the samples of *R. linearifolia*. The numbers on the top indicate the sample numbers (1‐30): 1-10 (BAU), 11- (QUR), 16-20 (MED), 21-25 (YT), 25-30 (KET). M—Thermo Scientific GeneRuler DNA Ladder Mix, (100–10,000 bp).


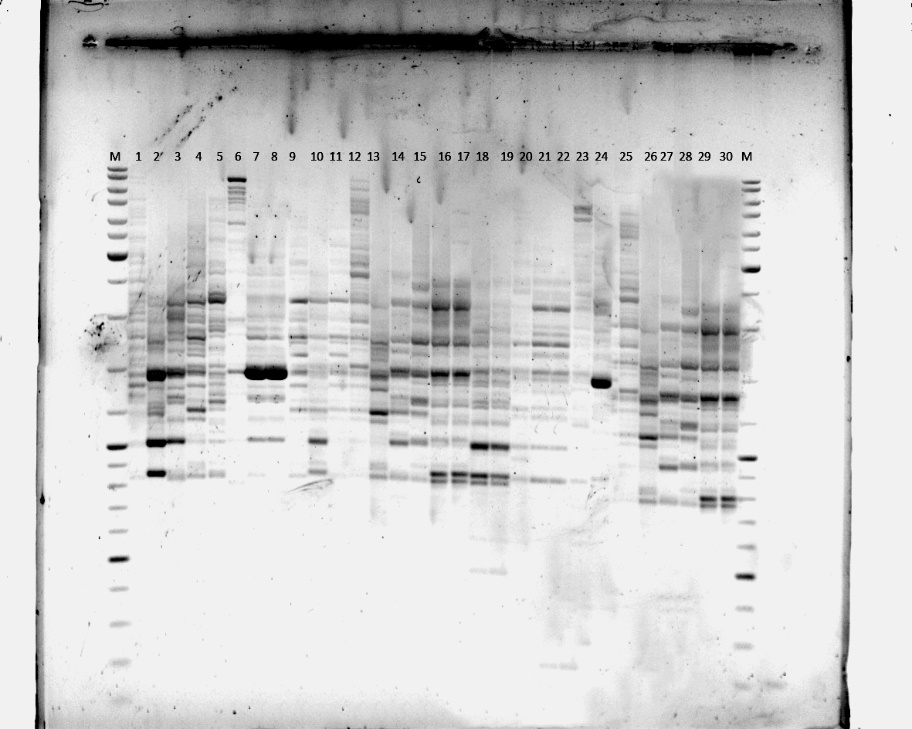


**Supplementary Figure 8.** The band profiles with iPBS primers 2397 for the samples of *R. linearifolia*. The numbers on the top indicate the sample numbers (1‐30): 1-10 (BAU), 11- (QUR), 16-20 (MED), 21-25 (YT), 25-30 (KET). M—Thermo Scientific GeneRuler DNA Ladder Mix, (100–10,000 bp).
